# Crystal structures of five (2-chloro­quinolin-3-yl)methyl ethers: supra­molecular assembly in one and two dimensions mediated by hydrogen bonding and π–π stacking

**DOI:** 10.1107/S2056989015008233

**Published:** 2015-05-13

**Authors:** Haliwana B. V. Sowmya, Tholappanavara H. Suresha Kumara, Nagendrappa Gopalpur, Jerry P. Jasinski, Sean P. Millikan, Hemmige S. Yathirajan, Christopher Glidewell

**Affiliations:** a PG Department of Chemistry, Jain University, 52 Bellary Road, Hebbal, Bangalore 560 024, India; b Department of Chemistry, UBDT College of Engineering (a Constituent College of VTU, Belagavi), Davanagere 577 004, India; c Department of Chemistry, Keene State College, 229 Main Street, Keene, NH 03435-2001, USA; d Department of Studies in Chemistry, University of Mysore, Manasagangotri, Mysore 570 006, India; e School of Chemistry, University of St Andrews, St Andrews, Fife KY16 9ST, Scotland

**Keywords:** crystal structure, 2-chloro­quinolines, mol­ecular conformation, hydrogen bonding, π–π stacking inter­actions

## Abstract

In the mol­ecules of the title compounds, methyl 5-bromo-2-[(2-chloro­quinolin-3-yl)meth­oxy]benzoate, C_18_H_13_BrClNO_3_, (I), methyl 5-bromo-2-[(2-chloro-6-methyl­quinolin-3-yl)meth­oxy]benzoate, C_19_H_15_BrClNO_3_, (II), methyl 2-[(2-chloro-6-methyl­quinolin-3-yl)meth­oxy]benzoate, C_19_H_16_ClNO_3_, (III), which crystallizes with *Z*′ = 4 in space group *P*2_1_2_1_2_1_, and 2-chloro-3-[(naphthalen-1-yl­oxy)meth­yl]quinoline, C_20_H_14_ClNO, (IV), the non-H atoms are nearly coplanar, but in {5-[(2-chloro­quinolin-3-yl)meth­oxy]-4-(hy­droxy­meth­yl)-6-methyl­pyridin-3-yl}methanol, C_18_H_17_ClN_2_O_3_, (V), the planes of the quinoline unit and of the unfused pyridine ring are almost parallel, although not coplanar. The mol­ecules of (I) are linked by two independent π–π stacking inter­actions to form chains, but there are no hydrogen bonds present in the structure. In (II), the mol­ecules are weakly linked into chains by a single type of π–π stacking inter­action. In (III), three of the four independent mol­ecules are linked by π–π stacking inter­actions but the other mol­ecule does not participate in such inter­actions. Weak C—H⋯O hydrogen bonds link the mol­ecules into three types of chains, two of which contain just one type of independent mol­ecule while the third type of chain contains two types of mol­ecule. The mol­ecules of (IV) are linked into chains by a C—H⋯π(arene) hydrogen bond, but π–π stacking inter­actions are absent. In (V), there is an intra­molecular O—H⋯O hydrogen bond, and mol­ecules are linked into sheets by a combination of O—H⋯N hydrogen bonds and π–π stacking inter­actions.

## Abstract 2

Five closely related (2-chloro­quinlin-3-yl)methyl ethers all exhibit different patterns of direction-specific inter­molecular inter­actions, leading to the formation of different types of chain in four of them and sheets in the fifth.

## Chemical context   

**Figure 1:**
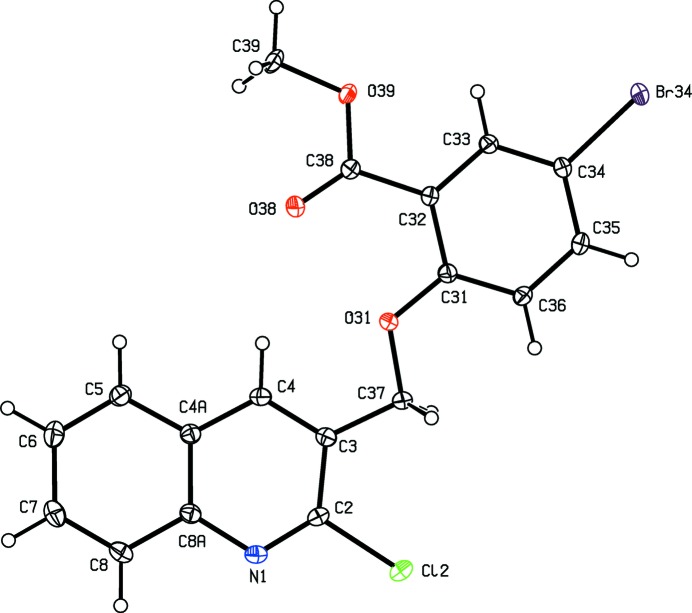
The mol­ecular structure of compound (I) showing the atom-labelling scheme. Displacement ellipsoids are drawn at the 30% probability level.

**Figure 2:**
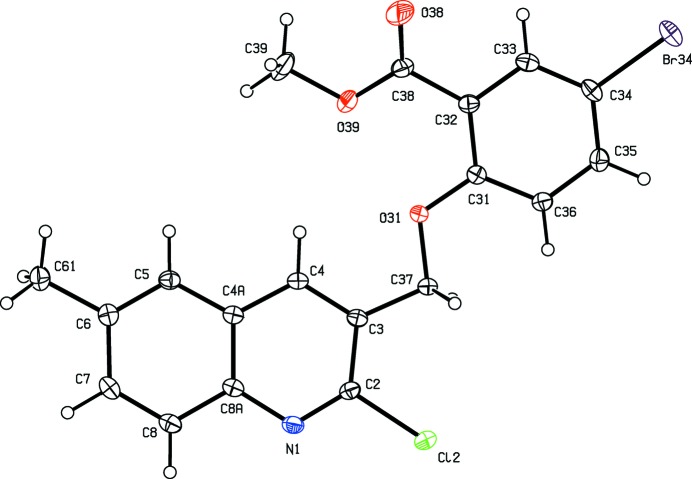
The mol­ecular structure of compound (II) showing the atom-labelling scheme. Displacement ellipsoids are drawn at the 30% probability level.

**Figure 3:**
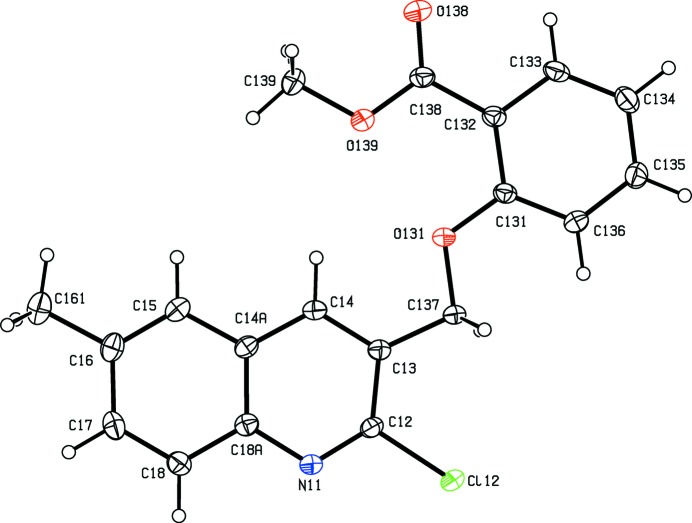
The structure of a type 1 mol­ecule of compound (III), showing the atom-labelling scheme. Displacement ellipsoids are drawn at the 30% probability level.

**Figure 7:**
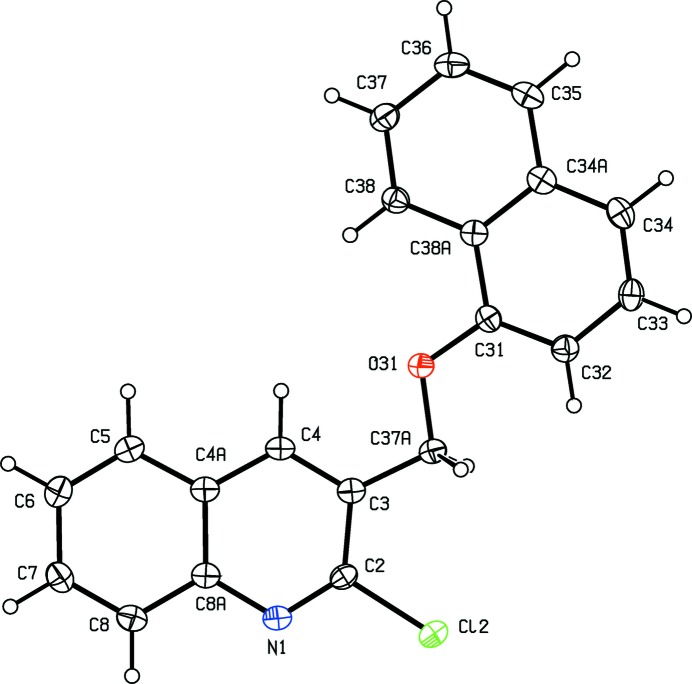
The mol­ecular structure of compound (IV) showing the atom-labelling scheme. Displacement ellipsoids are drawn at the 30% probability level.

**Figure 8:**
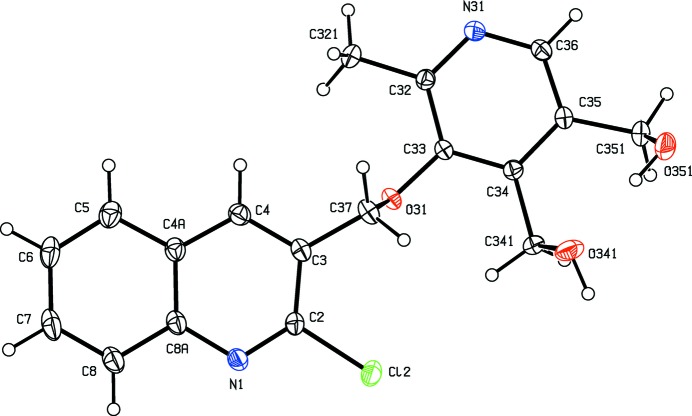
The mol­ecular structure of compound (V) showing the atom-labelling scheme. Displacement ellipsoids are drawn at the 30% probability level.

The quinoline nucleus occurs in a number of natural compounds, such as the Cinchona alkaloids, and many of these are pharmacologically active substances displaying a broad range of biological activity. Quinoline itself has been found to possess anti­malarial, anti-bacterial, anti­fungal, anthelminthic, cardiotonic, anti­convulsant, anti-inflammatory and analgesic activity (Marella *et al.*, 2013 ▸). The synthesis, reactions and biological applications of 2-chloro­quinoline-3-carbaldehydes have been reviewed (Abdel-Wahab *et al.*, 2012 ▸), and the structure of a simple reduction product (2-chloro­quinolin-3-yl)methanol, derived from the parent 2-chloro­quinoline-3-carbaldehyde, has been reported (Hathwar *et al.*, 2010 ▸). The structures of two related esters, [(2-chloro­quinolin-3-yl)methyl acetate and (2-chloro-6-methyl­quinolin-3-yl)methyl acetate], have also been reported recently along with a study of their radical-scavenging and anti­microbial activities (Tabassum *et al.*, 2014 ▸). Here we report the structures of five related ethers, namely methyl 5-bromo-2-[(2-chloro­quinolin-3-yl)meth­oxy]benzoate, (I) (Fig. 1 ▸), methyl 5-bromo-2-[(2-chloro-6-methyl­quinolin-3-yl)meth­oxy]benzoate, (II) (Fig. 2 ▸), methyl 2-[(2-chloro-6-methyl­quinolin-3-yl)meth­oxy]benzoate, (III) (Figs. 3 ▸–6 ▸ ▸ ▸), 2-chloro-3-[(naphthalen-1-yl­oxy)meth­yl]quinoline (IV) (Fig. 7 ▸) and {5-[(2-chloro­quinolin-3-yl)methoxy]-4-(hy­droxy­meth­yl)-6-methylpyridin-3-yl}methanol, (V) (Fig. 8 ▸). Compounds (I)–(V) are all of general type *Q*CH_2_O*R*, where *Q* represents a 2-chloro­quinolin-3-yl unit, which carries a 6-methyl substituent in compounds (II) and (III), although not in compounds (I), (IV) and (V), and where *R* represents a meth­oxy­carbonyl­phenyl unit in compounds (I)–(III), a 1-naphthyl unit in compound (IV), and a multiply-substituted pyridyl unit in compound (V). Compound (I)–(V) were all prepared by reaction of the corresponding chloro­methyl compounds *Q*CH_2_Cl with the appropriate hy­droxy compound *R*OH under basic conditions, with yields ranging from 86 to 97%.
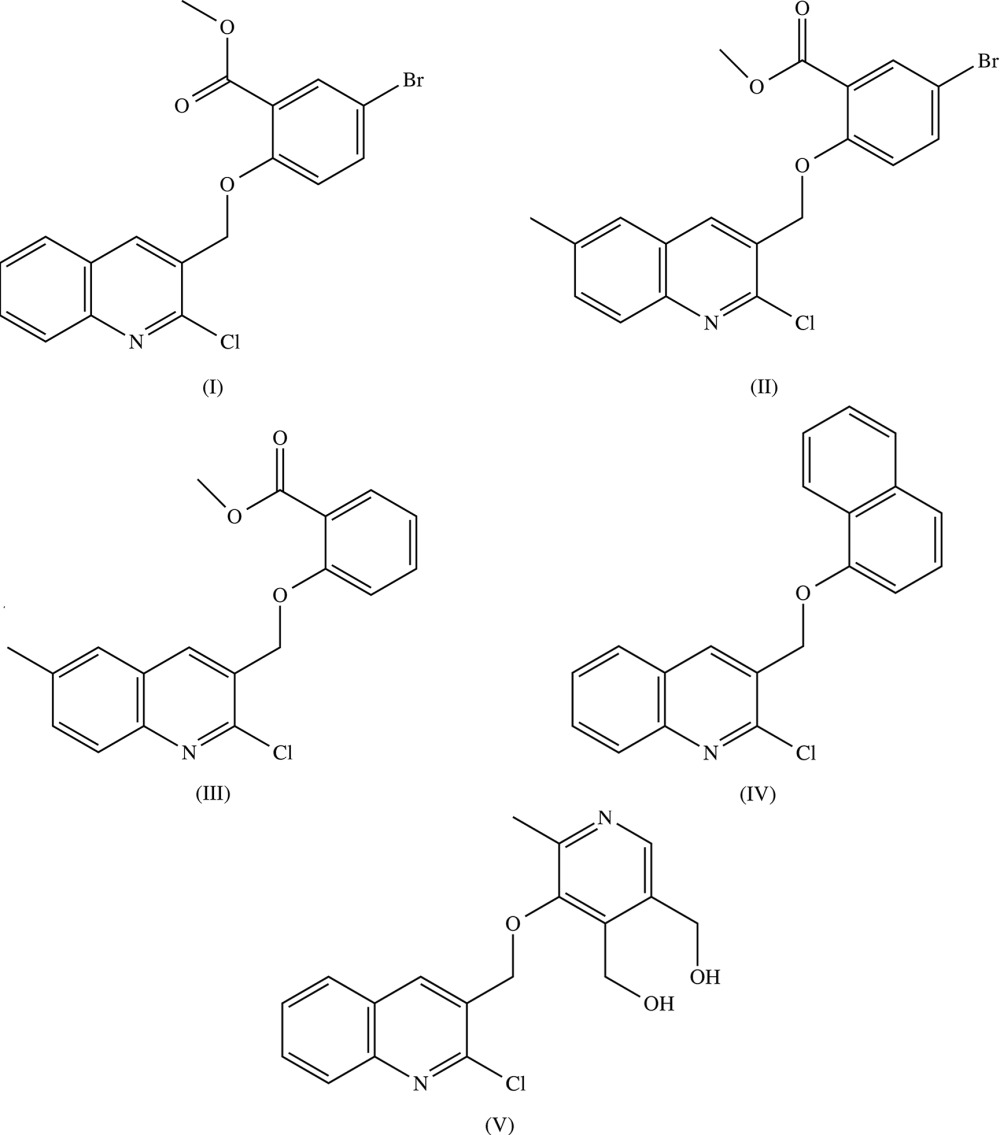


## Structural commentary   

As noted above, the mol­ecular constitutions of compounds (I)–(III) are very similar: those of compounds (I) and (II) differ only in the presence of a 6-methyl substituent in (II) which is absent from (I), while those of compounds (II) and (III) differ only in the presence of a bromo substituent in (II) which is absent from (III). Despite these close similarities, compounds (I)–(III) all crystallize in different space groups, *P*2_1_/*n* and Pbca, respectively, for (I) and (II), both with *Z*′ = 1, and *P*2_1_2_1_2_1_ with *Z*′ = 4 for (III). A search for possible additional crystallographic symmetry in compound (III) found none: comparison of the atomic coordinates for the Cl atoms within the selected asymmetric unit shows that while the *x*-coordinates of atoms Cl12 and Cl32 differ by *ca* 0.5 and their *z*-coordinates are almost identical, the *y*-coordinates of these two atoms differ by *ca* 0.13; similarly the *x*-coordinates of atoms Cl22 and Cl42 again differ by *ca* 0.5 but now the *y*-coordinates are almost identical, while the *z*-coordinates differ by *ca* 0.18. Hence it is not possible to identify even pseudosymmetry here. For compound (III), it will be convenient to refer to the mol­ecules containing atoms N11—N14 as mol­ecules of types 1–4, respectively. Compounds (IV) and (V) both crystallize with *Z*′ = 1, in space groups *P*2_1_ and *P*2_1_/*c*, respectively.

In compounds (I)–(III), the non-H atoms are almost co-planar, as shown by the relevant torsional and dihedral angles (Table 1 ▸). It is inter­esting to note that the orientation of the ester function in compound (I) differs from that in compounds (II) and (III) (Table 1 ▸ and Figs. 1 ▸–6 ▸ ▸ ▸ ▸ ▸): this difference may arise, at least in part, from the participation of the carbonyl O atom of the ester unit in short C—H⋯O inter­actions in all of the mol­ecules of compounds (II) and (III) but not in compound (I) (Table 2 ▸). The non-H atoms in compound (IV) are also nearly coplanar, with a dihedral angle between the mean planes of the quinoline and naphthalene units of 7.39 (12)°. By contrast, while the quinoline and pyridine units in compound (V) are nearly parallel (Fig. 8 ▸), with a dihedral angle between their mean planes of only 3.10 (9)°, they are by no means coplanar, as indicated by the values of the torsional angles C2—C3—C37—O31, 92.08 (18), C3—C37—O31—C33, 165.21 (13) and C37—O31—C33—C32, −90.17 (17)°. This again may perhaps be ascribed in part to the strong hydrogen bonds present in the crystal structure of (V) (Table 2 ▸).

None of the mol­ecules of compounds (I)–(V) exhibits any inter­nal symmetry and hence all are conformationally chiral. For compounds (I), (II) and (V), the centrosymmetric space groups accommodate equal numbers of the two conformational enanti­omers, but only one such enanti­omer is present in each crystal of compound (IV): the absolute configuration of the enanti­omer present in the crystal selected for data collection was established by means of the Flack *x* parameter (Flack, 1983 ▸), although this has no chemical significance. For compound (III), the value of the Flack *x* parameter gives evidence of partial inversion twinning.

## Supra­molecular inter­actions   

The supra­molecular assembly in compounds (I)–(V) is determined by a variety of direction-specific inter­molecular inter­actions, including both π–π stacking inter­actions and hydrogen bonds of C—H⋯N, C—H⋯O and C—H⋯π types, as well as O—H⋯N hydrogen bonds in compound (V) only. In compound (III), there are two fairly short inter­molecular C—H⋯N contacts involving C—H bonds from methyl groups bonded to the quinoline nucleus: not only are such bonds of low acidity, but these methyl groups are likely to be undergoing very rapid rotation about the adjacent C—C bonds (Riddell & Rogerson, 1996 ▸, 1997 ▸). When a group having local *C*_3_ symmetry, such as a methyl group, is directly bonded to a group having approximate local *C*_2_ symmetry, such as an aryl ring, the rotational barrier between these two groups is extremely low, of the order of J mol^−1^ rather than the usual kJ mol^−1^ (Naylor & Wilson, 1957 ▸; Tannenbaum *et al.*, 1956 ▸). Accordingly, these contacts in (III) are not regarded as having any structural significance. Likewise, the C—H⋯O contact in (V) involving the methyl group bonded to the unfused pyridine ring is not regarded as significant.

**Figure 9:**
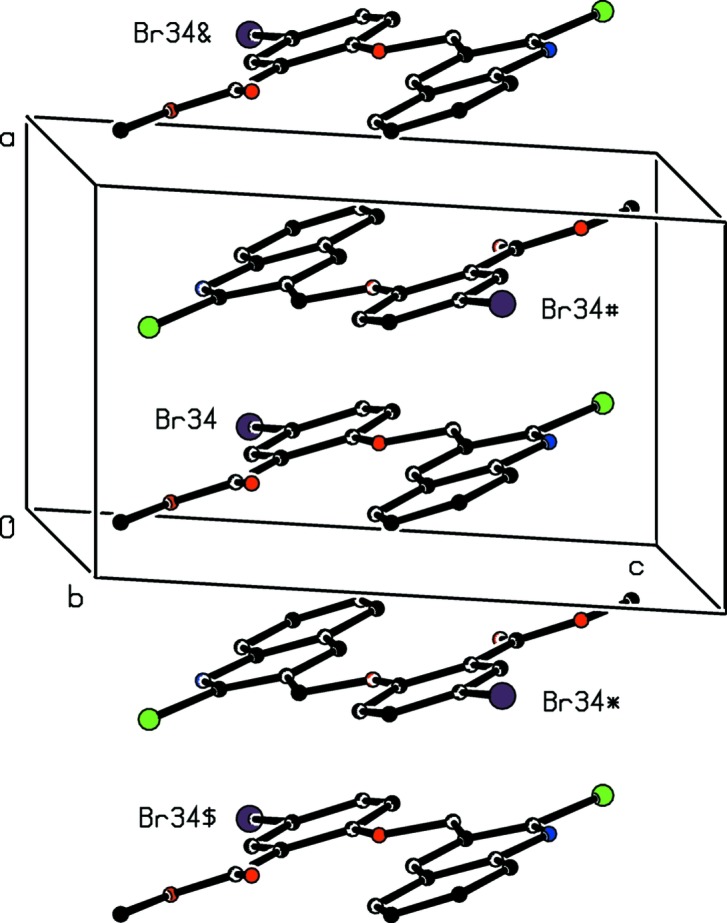
Part of the crystal structure of compound (I) showing the formation of a π-stacked chain along [100]. For the sake of clarity, H atoms have been omitted. Atoms marked with an asterisk (*), a hash (#), a dollar sign ($) or an ampersand (&) are at the symmetry positions (−*x*, −*y* + 1, −*z* + 1), (−*x* + 1, −*y* + 1, −*z* + 1), (*x* − 1, *y*, *z*) and (*x* + 1, *y*, *z*), respectively.

There are no hydrogen bonds of any kind in the crystal structure of compound (I), but mol­ecules are linked into chains by π–π stacking inter­actions. The fused aryl ring of the mol­ecule at (*x*, *y*, *z*) and the brominated ring of the mol­ecule at (−*x* + 1, −*y* + 1, −*z* + 1) make a dihedral angle of 1.04°; the ring centroid separation is 3.6168 (10) Å, and the shortest perpendicular distance from the centroid of one ring to the plane of the other is 3.4132 (6) Å, with a ring-centroid offset of *ca* 1.20 Å. For the heterocyclic ring at (*x*, *y*, *z*) and the brominated aryl ring at (−*x*, 1 − *y*, 1 − *z*), the corresponding values are 1.52 (9)°, 3.7454 (11) Å, 3.4357 (8) Å and *ca* 1.49 Å. The combination of these two stacking inter­actions links the mol­ecules of (I) into a chain running parallel to the [100] direction (Fig. 9 ▸). Two chains of this type pass through each unit cell but there are no direction-specific inter­actions between adjacent chains.

The only short C—H⋯O contact in the structure of compound (II) has a C—H⋯O angle of only 136° (Table 2 ▸), and so it is unlikely to be of major structural significance (Wood *et al.*, 2009 ▸). However, there is a weak π–π stacking inter­action between mol­ecules related by a 2_1_ screw axis. The pyridyl ring at (*x*, *y*, *z*) and the brominated aryl ring at (−*x* + 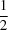, *y* + 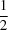, *z*) make a dihedral angle of 3.87 (10)°: the shortest perpendicular distance from the centroid of one ring to the plane of the other is 3.3816 (9) Å, but the ring-centroid separation is 3.882 (12), resulting in a ring-centroid offset of *ca* 1.78 Å. Thus there is only a very modest overlap of these rings and a consequently weak stacking inter­action: if this inter­action is, in fact, regarded as significant, it links the mol­ecules into a π-stacked chain running parallel to [010].

**Figure 10:**
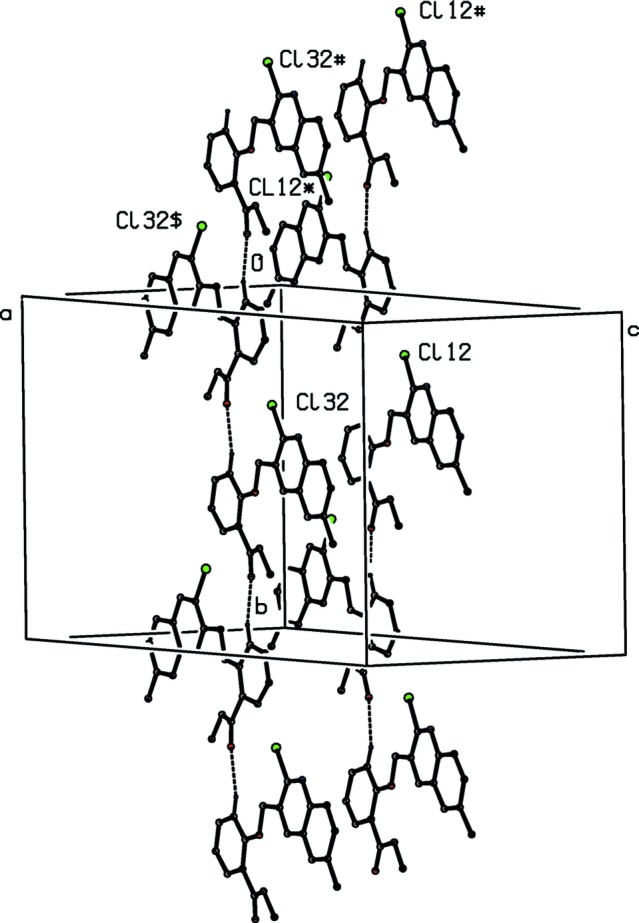
Part of the crystal structure of compound (III) showing the formation of two independent chains running parallel to the [010] direction and formed separately by the mol­ecules of types 1 and 3. For the sake of clarity, H atoms not involved in the motifs shown have been omitted. Atoms marked with an asterisk (*), a hash (#) or a dollar sign ($) are at the symmetry positions (−*x*, *y* − 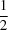, −*z* + 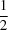), (*x*, *y* − 1, *z*) and (−*x* + 1, *y* − 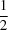, −*z +* + 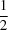), respectively.

**Figure 11:**
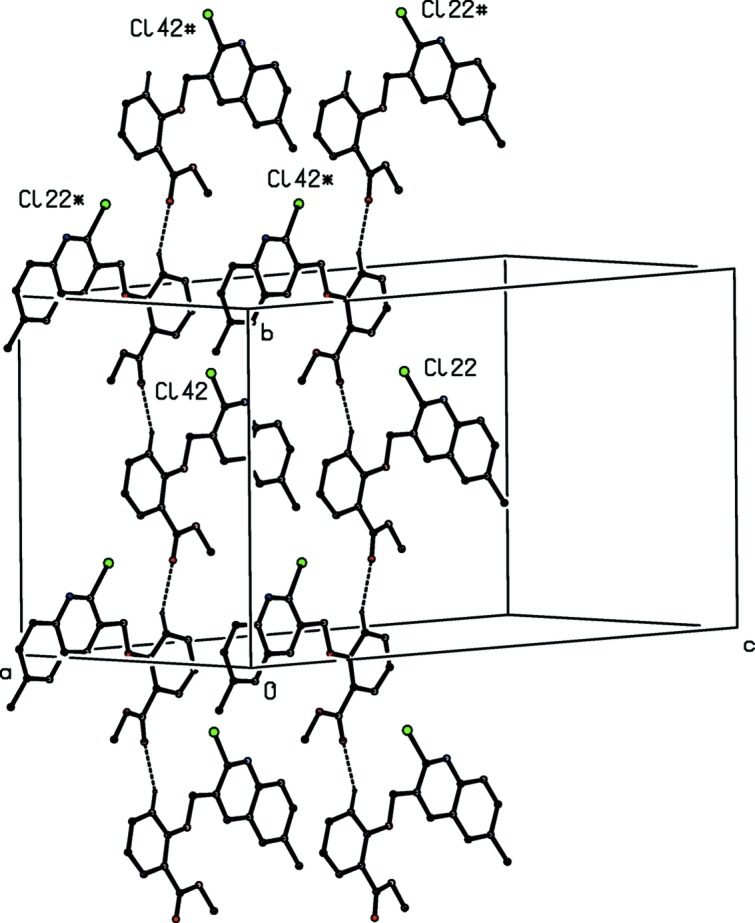
Part of the crystal structure of compound (III) showing the formation of a chain running parallel to the [010] direction and containing alternating mol­ecules of types 2 and 4. For the sake of clarity, H atoms not involved in the motifs shown have been omitted. Atoms marked with an asterisk (*) or a hash (#) are at the symmetry positions (−*x* + 1, *y* + 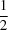, −*z* + 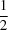) and (*x*, *y* + 1, *z*), respectively.

Within the selected asymmetric unit for compound (III), three of the four independent mol­ecules, those of types 2, 3 and 4 (*cf.* Figs. 3 ▸–6 ▸ ▸ ▸), are linked by two π–π stacking inter­actions, but the type 1 mol­ecule does not participate in any such inter­action. One of these stacking inter­actions involves the pyridyl ring of the type 2 mol­ecule and the fused aryl ring of the type 3 mol­ecule, while the other involves the pyridyl ring of the type 3 mol­ecule and the fused aryl ring of the type 4 mol­ecule. The dihedral angles between the ring planes within these two inter­actions are 3.11 (18) and 0.96 (7)°, respectively, the ring-centroid separations are 3.553 (2) Å and 3.544 (2) Å, and the shortest perpendicular distances from the centroid of one ring in each inter­action to the plane of the other ring are 3.4014 (15) and 3.3820 (15) Å, corresponding to ring-centroid offsets of *ca* 1.03 and *ca* 1.06 Å, respectively. The only short C—H⋯N contact within the crystal structure of compound (III) has an H⋯N distance which is not significantly less than the sum of the van der Waals radii, but there are four independent C—H⋯O hydrogen bonds present in the structure although all are probably weak as they have quite small C—H⋯O angles (Table 2 ▸). However, the pattern of these contacts is of inter­est as it precludes the possibility of any additional crystallographic symmetry in this structure where *Z*′ = 4. One of the C—H⋯O inter­actions involves only mol­ecules of type 1 which are related by the 2_1_ screw axis along (0, *y*, 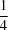), forming a *C*(6) (Bernstein *et al.*, 1995 ▸) running parallel to the [010] direction (Fig. 10 ▸); an entirely similar chain is formed by type 3 mol­ecules related to one another by the 2_1_ screw axis along (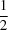, *y*, 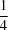). However, the mol­ecules of types 2 and 4 which are related by the 2_1_ screw axis along (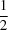, *y*, 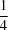) together form a 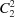
(12) chain parallel to [010] (Fig. 11 ▸), which runs anti­parallel to the chains formed by the mol­ecules of types 1 and 3. Hence the patterns of supra­molecular assembly in compounds (I)–(III), as well as their crystallization characteristics, show significant differences.

There are no hydrogen bonds of the C—H⋯N or C—H⋯O types in the crystal structure of compound (IV) and, despite the large number of independent aromatic rings, there are no π–π stacking inter­actions. The only direction-specific inter­molecular inter­action is a weak C—H⋯π(arene) contact involving mol­ecules related by translation.

**Figure 12:**
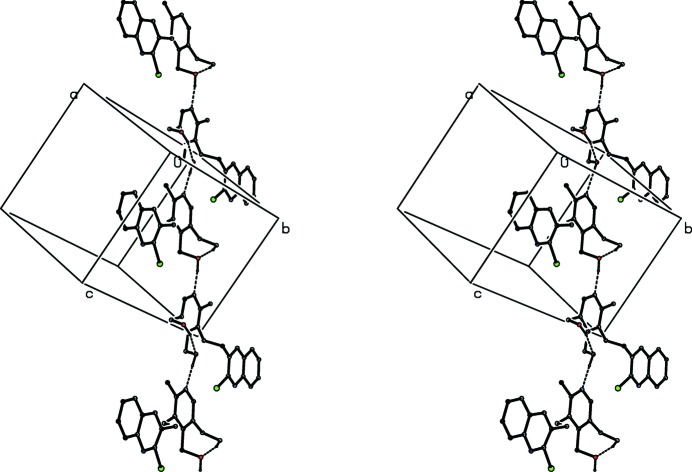
A stereoview of part of the crystal structure of compound (V) showing the formation of a *C*(7) chain formed by O—H⋯N hydrogen bonds and running parallel to [10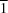]. For the sake of clarity, H atoms bonded to C atoms have been omitted.

**Figure 13:**
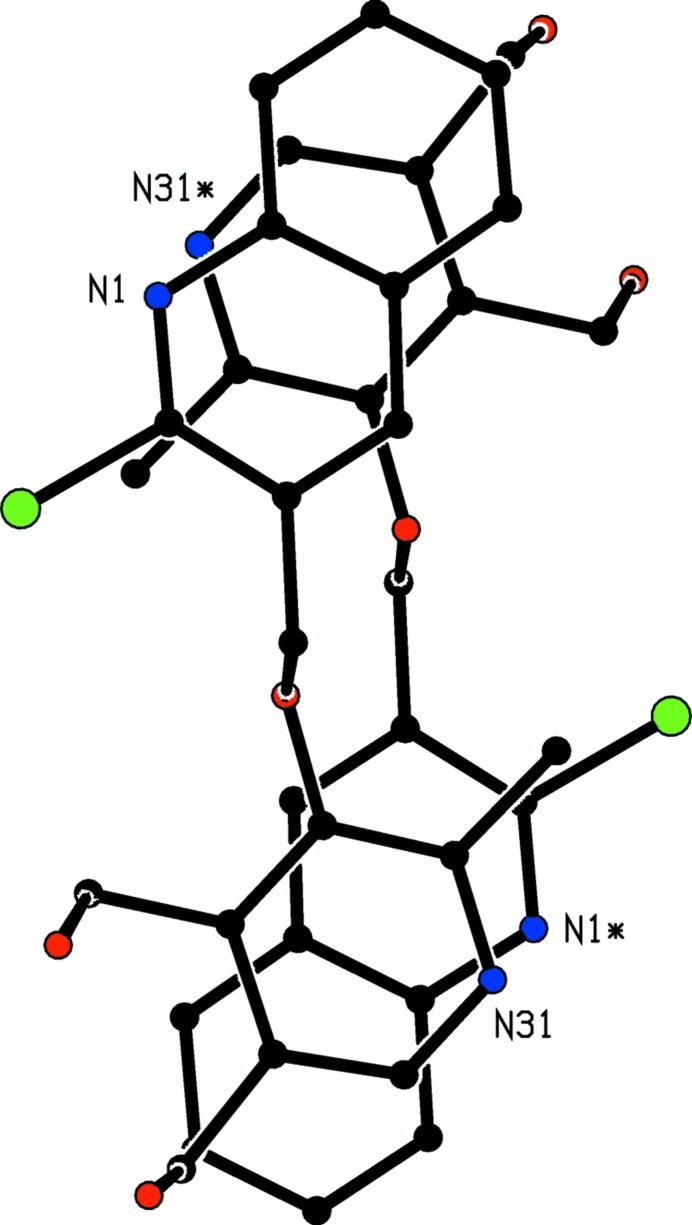
Part of the crystal structure of compound (V) showing the formation of a centrosymmetric π-stacked dimer. For the sake of clarity, H atoms have all been omitted. Atoms marked with an asterisk (*) are at the symmetry position (−*x* + 1, −*y* + 1, −*z* + 1).

**Figure 14:**
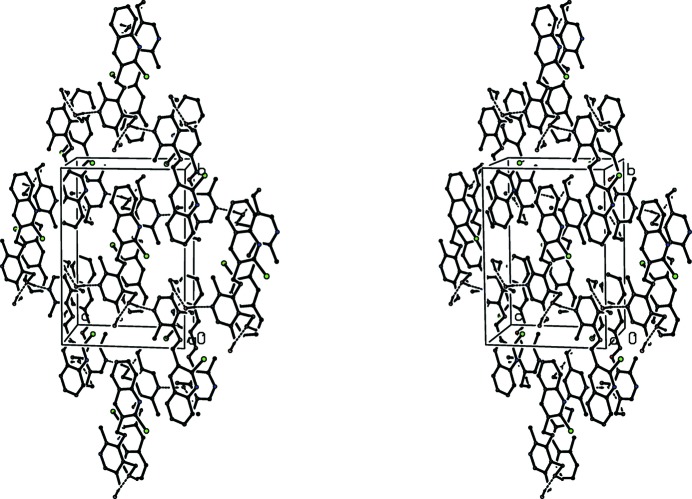
A stereoview of part of the crystal structure of compound (V) showing the formation of a π-stacked sheet of hydrogen bonded chains lying parallel to (101). For the sake of clarity, H atoms bonded to C atoms have been omitted.

The supra­molecular assembly in compound (V) is, however, rather more elaborate, resulting in part from the presence of additional hydrogen-bond donors and acceptors in the unfused pyridine unit. An intra­molecular O—H⋯O hydrogen bond (Table 2 ▸) gives rise to an *S*(7) (Bernstein *et al.*, 1995 ▸) motif, and an inter­molecular O—H⋯N hydrogen bond links mol­ecules related by the *n*-glide plane at *y* = 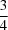, forming a *C*(7) chain running parallel to the [10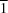] direction (Fig. 12 ▸). In addition, inversion-related pairs of mol­ecules are linked by π–π stacking inter­actions involving the unfused pyridine ring of one mol­ecule and the quinoline unit of the other (Fig. 13 ▸). Thus the unfused pyridine ring of the mol­ecule at (*x*, *y*, *z*) and the fused pyridine ring of the mol­ecule at (1 − *x*, 1 − *y*, 1 − *z*) make a dihedral angle of 4.43 (8)°: the ring-centroid separation is 3.7499 (9) Å and the shortest perpendicular distance from the centroid of one ring to the plane of the other is 3.5077 (7) Å, corresponding to a ring-centroid offset of *ca* 1.33 Å. For the unfused pyridyl ring at (*x*, *y*, *z*) and the fused aryl ring at (−*x* + 1, −*y* + 1, −*z* + 1) the corresponding values are 1.73 (8)°, 3.7333 (10) Å, 3.4637 (8) Å and *ca* 1.39 Å, respectively. The effect of the hydrogen-bonded chains is to link the π-stacked dimer centered at (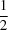, 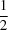, 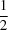) directly to the four symmetry-related dimers centred at (1, 0, 0), (1, 1, 0), (0, 0, 1) and (0, 1, 1), thus forming a sheet of π-stacked hydrogen-bonded chains lying parallel to (101) [Fig. 14 ▸].

## Database survey   

The structures of a number of fairly simple 2-chloro­quinolione derivatives related to compounds (I)–(V) have been reported in recent years. A structural study of a closely related group of six simply substituted 2-chloro­quinolines (Hathwar *et al.*, 2010 ▸) focused on supra­molecular aggregation *via* C—H⋯Cl hydrogen bonds and attractive Cl⋯Cl inter­actions. However, it must be pointed out firstly that it is now well established (Brammer *et al.*, 2001 ▸; Thallapally & Nangia, 2001 ▸) that Cl atoms bonded to C atoms are extremely poor acceptors of hydrogen bonds, even from strong donors such as O—H or N—H; and secondly, that for none of the compounds in this group were the shortest inter­molecular Cl⋯Cl distances less than the sum of the van der Waals radii (Bondi, 1964 ▸; Nyburg & Faerman, 1985 ▸; Rowland & Taylor, 1996 ▸): indeed, the concept of the van der Waals radius was nowhere mentioned by the original authors. Two of the six compounds in this group contained 3-hy­droxy­methyl substituents and, in each of these, the mol­ecules are linked into *C*(6) chains by means of O—H⋯N hydrogen bonds.

Mol­ecules of 2-[(2-chloro­quinolin-3-yl)(hy­droxy)meth­yl]acrylo­nitrile (Anuradha *et al.*, 2013*a* ▸) are also linked into *C*(6) chains by O—H⋯N hydrogen bonds, while in the closely related methyl 2-[(2-chloro­quinolin-3-yl)(hy­droxy)meth­yl]acrylate, where *Z*′ = 2 (Anuradha *et al.*, 2013*b* ▸), mol­ecules of one type are linked by O—H⋯O hydrogen bonds, again forming *C*(6) chains to which the mol­ecules of the second type are linked by O—H⋯N hydrogen bonds. Chains of *C*(6) type are formed also in *N*-{(2-chloro-3-quinlin­yl)meth­yl]-4-fluoro­aniline (Jasinski *et al.*, 2010 ▸), which is closely related to compounds (I)–(V) except that an amino linkage replaces the ether linkage in (I)–(V), so that the chains are built from N—H⋯N hydrogen bonds.

In the esters (2-choloroquinolin-3-yl)methyl acetate and (2-chloro-6-methyl­quinolin-3-yl)methyl acetate (Tabassum *et al.*, 2014 ▸), there are no strong hydrogen bond donors: in the methyl­ated compound, where *Z*′ = 2, the only hydrogen bond, of C—H⋯O type, links the two independent mol­ecules, while in the unmethyl­ated compound, the mol­ecules are linked into *C*(5) chains by C—H⋯N hydrogen bonds. In the structure of 2-chloro-3-(di­meth­oxy­meth­yl)-6-meth­oxy­quinoline (Chandrika *et al.*, 2015 ▸), there are no hydrogen bonds of any kind.

## Synthesis and crystallization   

For the synthesis of compounds (I)–(V), a mixture of 0.4 mmol of the appropriate quinoline derivative, 2-chloro-3-(chloro­meth­yl)quinoline for compounds (I), (IV) and (V) or 2-chloro-3-(chloro­meth­yl)-5-methyl­quinoline for compounds (II) and (III) and 0.4 mmol of the appropriate hy­droxy compound, methyl 5-bromo-2-hy­droxy­benzoate for (I) and (II), methyl 2-hy­droxy­benzoate for (III), 1-hy­droxy­naphthalene for (IV), or 3-hy­droxy-4,5-bis­(hy­droxy­meth­yl)-2-methyl­pyridinium chloride for (V), were dissolved in *N*,*N*-di­methyl­formamide (3–5 ml) together with potassium carbonate (2 mmol) and these mixtures were stirred at ambient temperature for 6–9 h, with monitoring by TLC. When each reaction was complete, ice-cold water (5 ml) was added and the resulting solid products were collected by filtration, washed with water and dried in air. Crystals suitable for single-crystal X-ray diffraction were obtained by slow evaporation, at ambient temperature and in the presence of air, of solutions in di­chloro­methane, with yields in the range 86–97%.

## Refinement   

Crystal data, data collection and structure refinement details are summarized in Table 3 ▸. All H atoms were located in difference Fourier maps. C-bound H atoms were then treated as riding atoms in geometrically idealized positions: C—H distances 0.95–0.99 Å with *U*_iso_(H) = 1.5*U*_eq_(C) for the methyl groups, which were permitted to rotate but not to tilt, and 1.2*U*_eq_(C) for other C-bound H atoms.

The H atoms bonded to O atoms in compound (V) were permitted to ride at the positions located in the difference Fourier map, with *U*_iso_(H) = 1.5*U*_eq_(O), giving O—H distances of 0.91 Å. For compound (III), the Flack *x* parameter (Flack, 1983 ▸) for the crystal selected for data collection was *x* = 0.161 (1) calculated (Parsons *et al.*, 2013 ▸) using 4617 quotients of type [(*I* ^+^)−(*I* ^−^)]/[(*I* ^+^)+(*I* ^−^)]. Use of the TWIN/BASF instructions in *SHELXL2014* (Sheldrick, 2015 ▸) gave a value for the twin fraction of 0.152 (16). For compound (IV), the absolute configuration of the conformational enanti­omer present in the crystal selected for data collection was established by means of the Flack *x* parameter calculated as *x* = −0.007 (18) by the standard method (Flack, 1983 ▸) and as *x* = 0.06 (2) calculated using 102 quotients of type [(*I* ^+^)−(*I* ^−^)]/[(*I* ^+^)+(*I* ^−^)].

## Supplementary Material

Crystal structure: contains datablock(s) global, I, II, III, IV, V. DOI: 10.1107/S2056989015008233/su5121sup1.cif

Structure factors: contains datablock(s) I. DOI: 10.1107/S2056989015008233/su5121Isup2.hkl

Structure factors: contains datablock(s) II. DOI: 10.1107/S2056989015008233/su5121IIsup3.hkl

Structure factors: contains datablock(s) III. DOI: 10.1107/S2056989015008233/su5121IIIsup4.hkl

Structure factors: contains datablock(s) IV. DOI: 10.1107/S2056989015008233/su5121IVsup5.hkl

Structure factors: contains datablock(s) V. DOI: 10.1107/S2056989015008233/su5121Vsup6.hkl

Supporting information file. DOI: 10.1107/S2056989015008233/su5121Isup7.cml

Supporting information file. DOI: 10.1107/S2056989015008233/su5121IIsup8.cml

Supporting information file. DOI: 10.1107/S2056989015008233/su5121IIIsup9.cml

Supporting information file. DOI: 10.1107/S2056989015008233/su5121IVsup10.cml

Supporting information file. DOI: 10.1107/S2056989015008233/su5121Vsup11.cml

CCDC references: 1061944, 992268, 992267, 1061945, 1061946

Additional supporting information:  crystallographic information; 3D view; checkCIF report

## Figures and Tables

**Figure 4:**
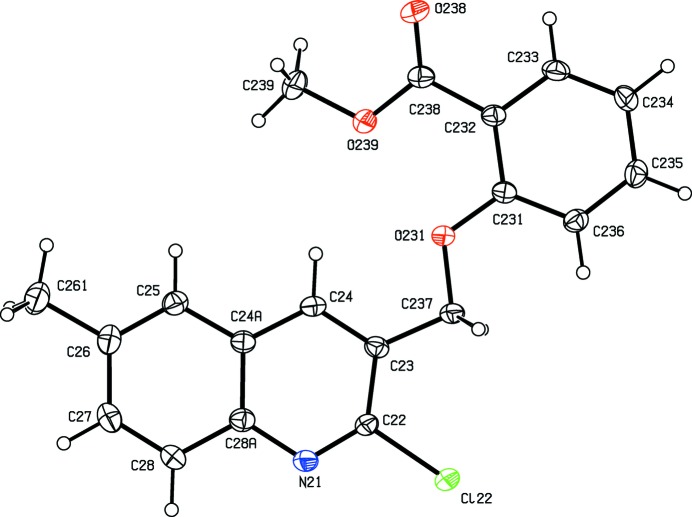
The structure of a type 2 mol­ecule of compound (III), showing the atom-labelling scheme. Displacement ellipsoids are drawn at the 30% probability level.

**Figure 5:**
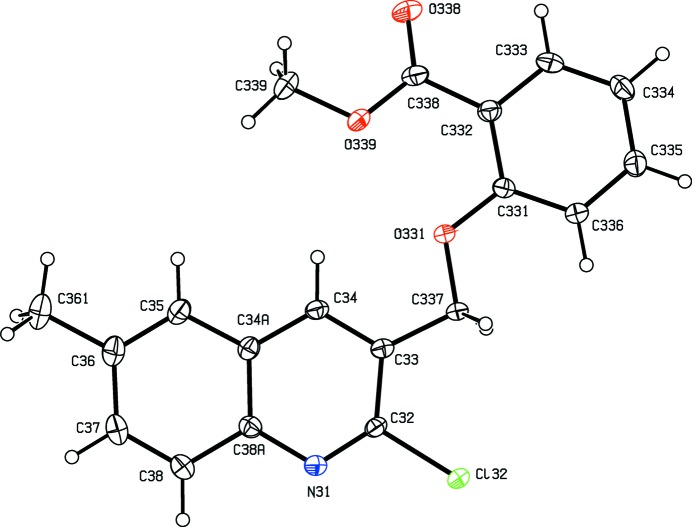
The structure of a type 3 mol­ecule of compound (III), showing the atom-labelling scheme. Displacement ellipsoids are drawn at the 30% probability level.

**Figure 6:**
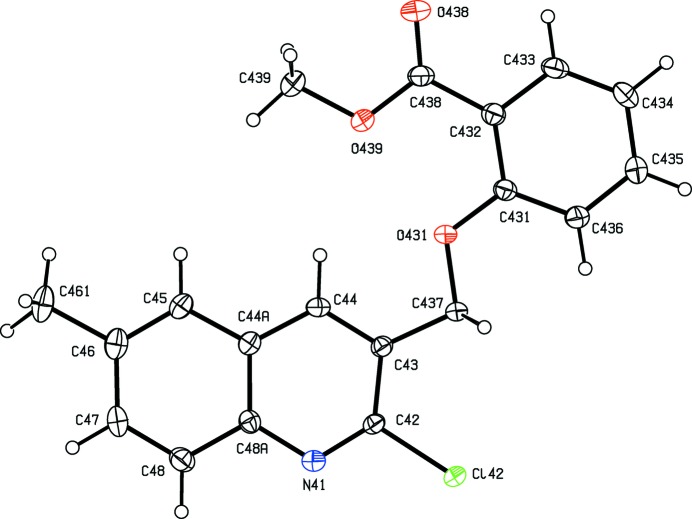
The structure of a type 4 mol­ecule of compound (III), showing the atom-labelling scheme. Displacement ellipsoids are drawn at the 30% probability level.

**Table 1:** Selected torsional and dihedral angles (°) for compounds (I)–(III) ‘Dihedral 1’ represents the dihedral angle between the mean planes of the quinoline and phenyl rings. ‘Dihedral 2’ represents the dihedral angle between the mean planes of the phenyl ring and the carboxyl unit.

Parameter	(I)	(II)	(III)			
*x*	nil	nil	1	2	3	4
C*x*2—C*x*3—C*x*37—O*x*31						
	−174.63 (17)	−176.93 (18)	−179.4 (3)	179.8 (3)	178.4 (3)	−177.6 (3)
C*x*3—C*x*37—O*x*31—C*x*31						
	−175.71 (16)	−179.57 (17)	177.2 (3)	−175.9 (3)	−178.9 (3)	176.4 (3)
C*x*37—O*x*31—C*x*31—C*x*32						
	173.73 (17)	−172.62 (18)	−176.8 (3)	174.5 (3)	177.7 (3)	−174.4 (3)
C*x*31—C*x*32—C*x*38—O*x*38						
	4.1 (3)	159.5 (3)	−177.5 (4)	166.4 (4)	−168.7 (4)	178.9 (4)
C*x*31—C*x*32—C*x*38—O*x*39						
	−177.01 (17)	−20.7 (3)	2.8 (6)	−14.8 (5)	12.7 (5)	−0.7 (5)
C*x*32—C*x*38—O*x*39—C*x*39						
	−175.77 (17)	−176.4 (2)	179.2 (3)	−176.4 (3)	178.3 (4)	180.0 (3)
Dihedral 1	0.66 (6)	10.72 (8)	5.44 (2)	4.18 (2)	3.825 (13)	5.55 (3)
Dihedral 2	4.27 (8)	19.25 (15)	2.52 (3)	14.66 (7)	12.29 (8)	1.78 (6)

**Table 2:** Hydrogen bonds and short inter­molecular contacts (Å, °) for compounds (II)–(V) *Cg*1, *Cg*2 and *Cg*3 are the centroids of rings C231–C236, C331–C336 and C31–C34,C34*A*,C38*A*, respectively.

Compound	*D*—H⋯*A*	*D*—H	H⋯A	*D*⋯*A*	*D*—H⋯*A*
(II)	C36—H36⋯O38^i^	0.95	2.53	3.277 (3)	136
(III)	C28—H28⋯N41^ii^	0.95	2.63	3.565 (5)	169
	C136—H136⋯O138^iii^	0.95	2.50	3.261 (4)	137
	C236—H236⋯O438^iv^	0.95	2.43	3.223 (4)	141
	C336—H336⋯O338^v^	0.95	2.46	3.238 (4)	139
	C436—H436⋯O238^iv^	0.95	2.51	3.254 (4)	136
	C337—H33*B*⋯*Cg*1	0.99	2.64	3.441 (4)	138
	C437—H43*A*⋯*Cg*2	0.99	2.64	3.446 (4)	138
(IV)	C37—H37*A*⋯*Cg*3^vi^	0.99	2.74	3.552 (3)	139
(V)	O341—H341⋯N31^vii^	0.91	1.81	2.7098 (19)	174
	O351—H351⋯O341	0.91	1.86	2.7209 (19)	158
	C4—H4⋯O351^viii^	0.95	2.60	3.374 (2)	139

Symmetry codes: (i) *x* + 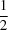, *y*, −*z* + 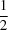; (ii) *x* − 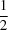, −*y* + 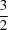, −*z* + 1; (iii) −*x*, *y* − 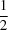, −*z* + 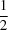; (iv) −*x* + 1, *y* + 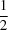, −*z* + 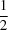; (v) −*x* + 1, *y* − 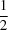, −*z* + 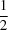; (vi) *x* − 1, *y*, *z*; (vii) *x* − 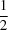, −*y* + 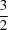, *z* + 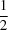; (viii) −*x* + 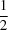, *y* − 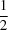, −*z* + 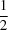.

**Table:** 

	(I)	(II)	(III)
Crystal data			
Chemical formula	C_18_H_13_BrClNO_3_	C_19_H_15_BrClNO_3_	C_19_H_16_ClNO_3_
*M*_r_	406.64	420.67	341.78
Crystal system, space group	Monoclinic, *P*2_1_/*n*	Orthorhombic, *P* *b* *c* *a*	Orthorhombic, *P*2_1_2_1_2_1_
Temperature (K)	173	173	173
*a*, *b*, *c* (Å)	7.3185 (4), 18.4177 (7), 11.7870 (5)	15.1920 (3), 11.98641 (19), 19.0307 (3)	13.5860 (3), 15.5857 (2), 30.9389 (5)
α, β, γ (°)	90, 93.609 (4), 90	90, 90, 90	90, 90, 90
*V* (Å^3^)	1585.62 (13)	3465.44 (10)	6551.2 (2)
*Z*	4	8	16
Radiation type	Mo *K*α	Cu *K*α	Cu *K*α
μ (mm^−1^)	2.78	4.81	2.21
Crystal size (mm)	0.44 × 0.23 × 0.12	0.24 × 0.16 × 0.08	0.48 × 0.26 × 0.14

Data collection			
Diffractometer	Agilent Eos Gemini	Agilent Eos Gemini	Agilent Eos Gemini
Absorption correction	Multi-scan (*SADABS*; Sheldrick, 2003 ▸)	Multi-scan (*SADABS*; Sheldrick, 2003 ▸)	Multi-scan (*SADABS*; Sheldrick, 2003 ▸)
*T*_min_, *T*_max_	0.335, 0.717	0.399, 0.680	0.472, 0.734
No. of measured, independent and observed [*I* > 2σ(*I*)] reflections	17735, 4612, 3682	21861, 3421, 3062	45901, 12840, 11257
*R*_int_	0.036	0.055	0.048
(sin θ/λ)_max_ (Å^−1^)	0.703	0.618	0.619

Refinement			
*R*[*F* ^2^ > 2σ(*F* ^2^)], *wR*(*F* ^2^), *S*	0.033, 0.071, 1.06	0.034, 0.093, 1.06	0.046, 0.129, 1.04
No. of reflections	4612	3421	12840
No. of parameters	218	229	874
No. of restraints	0	0	0
H-atom treatment	H-atom parameters constrained	H-atom parameters constrained	H-atom parameters constrained
Δρ_max_, Δρ_min_ (e Å^−3^)	0.42, −0.41	0.52, −0.49	0.42, −0.31
Absolute structure	–	–	Refined as an inversion twin
Absolute structure parameter	–	–	0.152 (16)

**Table:** 

	(IV)	(V)
Crystal data		
Chemical formula	C_20_H_14_ClNO	C_18_H_17_ClN_2_O_3_
*M*_r_	319.77	344.79
Crystal system, space group	Monoclinic, *P*2_1_	Monoclinic, *P*2_1_/*n*
Temperature (K)	173	173
*a*, *b*, *c* (Å)	5.3165 (3), 10.5098 (4), 13.6201 (7)	9.7866 (3), 15.3336 (4), 10.6570 (3)
α, β, γ (°)	90, 98.527 (5), 90	90, 92.381 (3), 90
*V* (Å^3^)	752.62 (6)	1597.85 (8)
*Z*	2	4
Radiation type	Cu *K*α	Cu *K*α
μ (mm^−1^)	2.27	2.29
Crystal size (mm)	0.34 × 0.10 × 0.08	0.42 × 0.38 × 0.32

Data collection		
Diffractometer	Agilent Eos Gemini	Agilent Eos Gemini
Absorption correction	Multi-scan (*SADABS*; Sheldrick, 2003 ▸)	Multi-scan (*SADABS*; Sheldrick, 2003 ▸)
*T*_min_, *T*_max_	0.551, 0.834	0.375, 0.481
No. of measured, independent and observed [*I* > 2σ(*I*)] reflections	4606, 2014, 1938	9423, 3112, 2764
*R*_int_	0.029	0.043
(sin θ/λ)_max_ (Å^−1^)	0.619	0.618

Refinement		
*R*[*F* ^2^ > 2σ(*F* ^2^)], *wR*(*F* ^2^), *S*	0.033, 0.090, 1.08	0.045, 0.127, 1.05
No. of reflections	2014	3112
No. of parameters	208	219
No. of restraints	1	0
H-atom treatment	H-atom parameters constrained	H-atom parameters constrained
Δρ_max_, Δρ_min_ (e Å^−3^)	0.22, −0.19	0.32, −0.25
Absolute structure	Classical Flack method preferred over Parsons because s.u. lower	
Absolute structure parameter	−0.007 (18)	–

Computer programs: *CrysAlis PRO* and *CrysAlis RED* (Agilent, 2012 ▸), *SHELXS97* (Sheldrick, 2008 ▸), *SHELXL2014* (Sheldrick, 2015 ▸) and *PLATON* (Spek, 2009 ▸).

## supplementary crystallographic information

### (I) Methyl 5-bromo-2-[(2-chloroquinolin-3-yl)methoxy]benzoate Crystal data

**Table:**

C_18_H_13_BrClNO_3_	*F*(000) = 816
*M**_r_* = 406.64	*D*_x_ = 1.703 Mg m^−^^3^
Monoclinic, *P*2_1_/*n*	Mo *K*α radiation, λ = 0.71073 Å
*a* = 7.3185 (4) Å	Cell parameters from 5443 reflections
*b* = 18.4177 (7) Å	θ = 3.0–33.0°
*c* = 11.7870 (5) Å	µ = 2.78 mm^−^^1^
β = 93.609 (4)°	*T* = 173 K
*V* = 1585.62 (13) Å^3^	Plate, colourles
*Z* = 4	0.44 × 0.23 × 0.12 mm

### (I) Methyl 5-bromo-2-[(2-chloroquinolin-3-yl)methoxy]benzoate Data collection

**Table:**

Agilent Eos Gemini diffractometer	3682 reflections with *I* > 2σ(*I*)
Radiation source: Enhance (Mo) X-ray Source	*R*_int_ = 0.036
ω scans	θ_max_ = 30.0°, θ_min_ = 3.0°
Absorption correction: multi-scan (*SADABS*; Sheldrick, 2003)	*h* = −10→8
*T*_min_ = 0.335, *T*_max_ = 0.717	*k* = −24→25
17735 measured reflections	*l* = −16→16
4612 independent reflections	

### (I) Methyl 5-bromo-2-[(2-chloroquinolin-3-yl)methoxy]benzoate Refinement

**Table:**

Refinement on *F*^2^	0 restraints
Least-squares matrix: full	Hydrogen site location: inferred from neighbouring sites
*R*[*F*^2^ > 2σ(*F*^2^)] = 0.033	H-atom parameters constrained
*wR*(*F*^2^) = 0.071	*w* = 1/[σ^2^(*F*_o_^2^) + (0.0288*P*)^2^ + 0.6015*P*] where *P* = (*F*_o_^2^ + 2*F*_c_^2^)/3
*S* = 1.06	(Δ/σ)_max_ = 0.001
4612 reflections	Δρ_max_ = 0.42 e Å^−^^3^
218 parameters	Δρ_min_ = −0.41 e Å^−^^3^

### (I) Methyl 5-bromo-2-[(2-chloroquinolin-3-yl)methoxy]benzoate Special details

**Table:**

Geometry. All e.s.d.'s (except the e.s.d. in the dihedral angle between two l.s. planes) are estimated using the full covariance matrix. The cell e.s.d.'s are taken into account individually in the estimation of e.s.d.'s in distances, angles and torsion angles; correlations between e.s.d.'s in cell parameters are only used when they are defined by crystal symmetry. An approximate (isotropic) treatment of cell e.s.d.'s is used for estimating e.s.d.'s involving l.s. planes.

### (I) Methyl 5-bromo-2-[(2-chloroquinolin-3-yl)methoxy]benzoate Fractional atomic coordinates and isotropic or equivalent isotropic displacement parameters (Å^2^)

**Table:**

	*x*	*y*	*z*	*U*_iso_*/*U*_eq_	
N1	0.3544 (2)	0.64638 (8)	0.75859 (12)	0.0228 (3)	
C2	0.3622 (2)	0.57729 (10)	0.74061 (14)	0.0218 (4)	
Cl2	0.44084 (7)	0.52448 (3)	0.85755 (4)	0.03157 (12)	
C3	0.3125 (2)	0.54040 (10)	0.63773 (15)	0.0209 (4)	
C4	0.2421 (2)	0.58250 (10)	0.55035 (15)	0.0219 (4)	
H4	0.2034	0.5606	0.4799	0.026*	
C4A	0.2265 (2)	0.65814 (10)	0.56389 (15)	0.0209 (4)	
C5	0.1545 (3)	0.70490 (11)	0.47672 (16)	0.0257 (4)	
H5	0.1115	0.6851	0.4055	0.031*	
C6	0.1462 (3)	0.77791 (11)	0.49390 (17)	0.0303 (4)	
H6	0.0952	0.8087	0.4354	0.036*	
C7	0.2134 (3)	0.80793 (11)	0.59853 (18)	0.0315 (4)	
H7	0.2101	0.8590	0.6093	0.038*	
C8	0.2826 (3)	0.76456 (11)	0.68391 (17)	0.0276 (4)	
H8	0.3271	0.7855	0.7539	0.033*	
C8A	0.2888 (2)	0.68865 (10)	0.66944 (14)	0.0212 (4)	
C37	0.3404 (3)	0.46017 (10)	0.62654 (15)	0.0246 (4)	
H37A	0.4696	0.4471	0.6467	0.030*	
H37B	0.2612	0.4333	0.6772	0.030*	
O31	0.2926 (2)	0.44326 (7)	0.51108 (10)	0.0275 (3)	
C31	0.2926 (2)	0.37331 (9)	0.47662 (14)	0.0203 (3)	
C32	0.2264 (2)	0.35949 (9)	0.36438 (14)	0.0191 (3)	
C33	0.2199 (2)	0.28798 (9)	0.32599 (14)	0.0203 (3)	
H33	0.1751	0.2779	0.2502	0.024*	
C34	0.2772 (3)	0.23189 (9)	0.39624 (15)	0.0212 (4)	
Br34	0.26437 (3)	0.13590 (2)	0.33927 (2)	0.03066 (7)	
C35	0.3456 (3)	0.24530 (10)	0.50667 (15)	0.0235 (4)	
H35	0.3860	0.2064	0.5548	0.028*	
C36	0.3543 (3)	0.31595 (10)	0.54578 (15)	0.0224 (4)	
H36	0.4030	0.3255	0.6209	0.027*	
C38	0.1676 (2)	0.41935 (10)	0.28586 (14)	0.0217 (4)	
O38	0.1774 (2)	0.48297 (8)	0.30438 (12)	0.0415 (4)	
O39	0.1018 (2)	0.39288 (7)	0.18579 (11)	0.0361 (4)	
C39	0.0528 (3)	0.44627 (11)	0.10042 (18)	0.0380 (5)	
H39A	−0.0067	0.4223	0.0336	0.057*	
H39B	0.1633	0.4714	0.0786	0.057*	
H39C	−0.0317	0.4815	0.1308	0.057*	

### (I) Methyl 5-bromo-2-[(2-chloroquinolin-3-yl)methoxy]benzoate Atomic displacement parameters (Å^2^)

**Table:**

	*U*^11^	*U*^22^	*U*^33^	*U*^12^	*U*^13^	*U*^23^
N1	0.0241 (8)	0.0260 (8)	0.0181 (7)	−0.0004 (6)	0.0007 (6)	−0.0038 (6)
C2	0.0222 (9)	0.0266 (9)	0.0164 (8)	0.0002 (7)	−0.0006 (7)	0.0005 (7)
Cl2	0.0414 (3)	0.0328 (3)	0.0196 (2)	0.0036 (2)	−0.00517 (19)	0.00316 (18)
C3	0.0220 (9)	0.0205 (9)	0.0202 (8)	−0.0018 (7)	0.0021 (7)	−0.0023 (7)
C4	0.0240 (9)	0.0237 (9)	0.0178 (8)	−0.0012 (7)	0.0002 (7)	−0.0028 (7)
C4A	0.0207 (9)	0.0222 (9)	0.0201 (8)	0.0005 (7)	0.0031 (7)	−0.0006 (7)
C5	0.0272 (10)	0.0282 (10)	0.0215 (8)	0.0036 (8)	0.0006 (7)	0.0006 (7)
C6	0.0314 (11)	0.0278 (10)	0.0320 (10)	0.0073 (8)	0.0048 (8)	0.0068 (8)
C7	0.0334 (11)	0.0228 (10)	0.0390 (11)	0.0045 (8)	0.0077 (9)	−0.0024 (8)
C8	0.0304 (11)	0.0242 (10)	0.0282 (9)	0.0009 (8)	0.0034 (8)	−0.0079 (8)
C8A	0.0199 (9)	0.0227 (9)	0.0213 (8)	0.0014 (7)	0.0036 (7)	−0.0029 (7)
C37	0.0354 (11)	0.0211 (9)	0.0170 (8)	0.0003 (7)	−0.0022 (7)	−0.0010 (7)
O31	0.0467 (9)	0.0168 (6)	0.0181 (6)	0.0019 (6)	−0.0048 (6)	−0.0020 (5)
C31	0.0231 (9)	0.0188 (9)	0.0191 (8)	−0.0002 (7)	0.0020 (7)	0.0000 (6)
C32	0.0215 (9)	0.0172 (8)	0.0186 (8)	0.0003 (6)	0.0009 (6)	0.0019 (6)
C33	0.0228 (9)	0.0187 (8)	0.0193 (8)	−0.0016 (7)	0.0003 (7)	0.0000 (6)
C34	0.0250 (9)	0.0158 (8)	0.0227 (8)	−0.0012 (6)	0.0008 (7)	0.0006 (7)
Br34	0.04557 (13)	0.01513 (9)	0.03011 (11)	−0.00002 (8)	−0.00682 (8)	−0.00001 (7)
C35	0.0284 (10)	0.0198 (9)	0.0224 (9)	0.0007 (7)	0.0010 (7)	0.0058 (7)
C36	0.0280 (10)	0.0209 (9)	0.0178 (8)	−0.0014 (7)	−0.0013 (7)	0.0026 (7)
C38	0.0247 (9)	0.0207 (9)	0.0197 (8)	0.0003 (7)	0.0011 (7)	0.0010 (7)
O38	0.0816 (12)	0.0175 (7)	0.0240 (7)	0.0027 (7)	−0.0074 (7)	0.0014 (6)
O39	0.0601 (10)	0.0178 (7)	0.0272 (7)	−0.0043 (6)	−0.0220 (7)	0.0050 (6)
C39	0.0540 (14)	0.0263 (11)	0.0307 (10)	−0.0027 (9)	−0.0207 (10)	0.0108 (8)

### (I) Methyl 5-bromo-2-[(2-chloroquinolin-3-yl)methoxy]benzoate Geometric parameters (Å, º)

**Table:**

N1—C2	1.292 (2)	C37—H37B	0.9900
N1—C8A	1.370 (2)	O31—C31	1.351 (2)
C2—C3	1.417 (2)	C31—C36	1.392 (2)
C2—Cl2	1.7542 (18)	C31—C32	1.403 (2)
C3—C4	1.364 (2)	C32—C33	1.393 (2)
C3—C37	1.499 (2)	C32—C38	1.486 (2)
C4—C4A	1.408 (3)	C33—C34	1.373 (2)
C4—H4	0.9500	C33—H33	0.9500
C4A—C8A	1.414 (2)	C34—C35	1.387 (2)
C4A—C5	1.417 (3)	C34—Br34	1.8913 (18)
C5—C6	1.362 (3)	C35—C36	1.380 (3)
C5—H5	0.9500	C35—H35	0.9500
C6—C7	1.411 (3)	C36—H36	0.9500
C6—H6	0.9500	C38—O38	1.193 (2)
C7—C8	1.358 (3)	C38—O39	1.338 (2)
C7—H7	0.9500	O39—C39	1.436 (2)
C8—C8A	1.410 (3)	C39—H39A	0.9800
C8—H8	0.9500	C39—H39B	0.9800
C37—O31	1.418 (2)	C39—H39C	0.9800
C37—H37A	0.9900		
			
C2—N1—C8A	116.80 (15)	C3—C37—H37B	110.6
N1—C2—C3	126.94 (16)	H37A—C37—H37B	108.7
N1—C2—Cl2	115.65 (13)	C31—O31—C37	119.56 (14)
C3—C2—Cl2	117.41 (14)	O31—C31—C36	123.63 (15)
C4—C3—C2	115.97 (16)	O31—C31—C32	116.73 (15)
C4—C3—C37	122.75 (16)	C36—C31—C32	119.63 (16)
C2—C3—C37	121.27 (16)	C33—C32—C31	118.73 (15)
C3—C4—C4A	120.43 (16)	C33—C32—C38	119.78 (15)
C3—C4—H4	119.8	C31—C32—C38	121.48 (15)
C4A—C4—H4	119.8	C34—C33—C32	120.84 (16)
C4—C4A—C8A	117.96 (16)	C34—C33—H33	119.6
C4—C4A—C5	123.28 (16)	C32—C33—H33	119.6
C8A—C4A—C5	118.75 (17)	C33—C34—C35	120.69 (16)
C6—C5—C4A	120.70 (18)	C33—C34—Br34	118.86 (13)
C6—C5—H5	119.7	C35—C34—Br34	120.44 (13)
C4A—C5—H5	119.7	C36—C35—C34	119.18 (16)
C5—C6—C7	120.03 (18)	C36—C35—H35	120.4
C5—C6—H6	120.0	C34—C35—H35	120.4
C7—C6—H6	120.0	C35—C36—C31	120.88 (16)
C8—C7—C6	120.69 (19)	C35—C36—H36	119.6
C8—C7—H7	119.7	C31—C36—H36	119.6
C6—C7—H7	119.7	O38—C38—O39	122.23 (16)
C7—C8—C8A	120.48 (18)	O38—C38—C32	127.08 (16)
C7—C8—H8	119.8	O39—C38—C32	110.68 (15)
C8A—C8—H8	119.8	C38—O39—C39	115.37 (15)
N1—C8A—C8	118.90 (16)	O39—C39—H39A	109.5
N1—C8A—C4A	121.80 (16)	O39—C39—H39B	109.5
C8—C8A—C4A	119.31 (17)	H39A—C39—H39B	109.5
O31—C37—C3	105.92 (14)	O39—C39—H39C	109.5
O31—C37—H37A	110.6	H39A—C39—H39C	109.5
C3—C37—H37A	110.6	H39B—C39—H39C	109.5
O31—C37—H37B	110.6		
			
C8A—N1—C2—C3	1.0 (3)	C2—C3—C37—O31	−174.63 (17)
C8A—N1—C2—Cl2	−178.33 (13)	C3—C37—O31—C31	−175.71 (16)
N1—C2—C3—C4	−2.9 (3)	C37—O31—C31—C36	−6.4 (3)
Cl2—C2—C3—C4	176.39 (14)	C37—O31—C31—C32	173.73 (17)
N1—C2—C3—C37	175.89 (19)	O31—C31—C32—C33	−178.50 (16)
Cl2—C2—C3—C37	−4.8 (2)	C36—C31—C32—C33	1.6 (3)
C2—C3—C4—C4A	1.6 (3)	O31—C31—C32—C38	2.8 (3)
C37—C3—C4—C4A	−177.14 (17)	C36—C31—C32—C38	−177.03 (17)
C3—C4—C4A—C8A	1.1 (3)	C31—C32—C33—C34	−0.1 (3)
C3—C4—C4A—C5	−179.93 (18)	C38—C32—C33—C34	178.58 (17)
C4—C4A—C5—C6	−178.59 (19)	C32—C33—C34—C35	−1.0 (3)
C8A—C4A—C5—C6	0.3 (3)	C32—C33—C34—Br34	179.85 (14)
C4A—C5—C6—C7	1.4 (3)	C33—C34—C35—C36	0.5 (3)
C5—C6—C7—C8	−1.6 (3)	Br34—C34—C35—C36	179.65 (14)
C6—C7—C8—C8A	0.1 (3)	C34—C35—C36—C31	1.1 (3)
C2—N1—C8A—C8	−178.21 (17)	O31—C31—C36—C35	178.00 (17)
C2—N1—C8A—C4A	2.2 (3)	C32—C31—C36—C35	−2.2 (3)
C7—C8—C8A—N1	−178.03 (18)	C33—C32—C38—O38	−174.5 (2)
C7—C8—C8A—C4A	1.6 (3)	C31—C32—C38—O38	4.1 (3)
C4—C4A—C8A—N1	−3.2 (3)	C33—C32—C38—O39	4.3 (2)
C5—C4A—C8A—N1	177.82 (17)	C31—C32—C38—O39	−177.01 (17)
C4—C4A—C8A—C8	177.18 (17)	O38—C38—O39—C39	3.1 (3)
C5—C4A—C8A—C8	−1.8 (3)	C32—C38—O39—C39	−175.77 (17)
C4—C3—C37—O31	4.1 (3)		

### (II) Methyl 5-bromo-2-[(2-chloro-6-methylquinolin-3-yl)methoxy]benzoate Crystal data

**Table:**

C_19_H_15_BrClNO_3_	*D*_x_ = 1.613 Mg m^−^^3^
*M**_r_* = 420.67	Cu *K*α radiation, λ = 1.54184 Å
Orthorhombic, *P**b**c**a*	Cell parameters from 3421 reflections
*a* = 15.1920 (3) Å	θ = 4.7–72.5°
*b* = 11.98641 (19) Å	µ = 4.81 mm^−^^1^
*c* = 19.0307 (3) Å	*T* = 173 K
*V* = 3465.44 (10) Å^3^	Block, colourless
*Z* = 8	0.24 × 0.16 × 0.08 mm
*F*(000) = 1696	

### (II) Methyl 5-bromo-2-[(2-chloro-6-methylquinolin-3-yl)methoxy]benzoate Data collection

**Table:**

Agilent Eos Gemini diffractometer	3062 reflections with *I* > 2σ(*I*)
Radiation source: Enhance (Cu) X-ray Source	*R*_int_ = 0.055
ω scans	θ_max_ = 72.5°, θ_min_ = 4.7°
Absorption correction: multi-scan (*SADABS*; Sheldrick, 2003)	*h* = −18→18
*T*_min_ = 0.399, *T*_max_ = 0.680	*k* = −14→12
21861 measured reflections	*l* = −23→18
3421 independent reflections	

### (II) Methyl 5-bromo-2-[(2-chloro-6-methylquinolin-3-yl)methoxy]benzoate Refinement

**Table:**

Refinement on *F*^2^	Hydrogen site location: inferred from neighbouring sites
Least-squares matrix: full	H-atom parameters constrained
*R*[*F*^2^ > 2σ(*F*^2^)] = 0.034	*w* = 1/[σ^2^(*F*_o_^2^) + (0.0523*P*)^2^ + 1.8465*P*] where *P* = (*F*_o_^2^ + 2*F*_c_^2^)/3
*wR*(*F*^2^) = 0.093	(Δ/σ)_max_ < 0.001
*S* = 1.06	Δρ_max_ = 0.52 e Å^−^^3^
3421 reflections	Δρ_min_ = −0.49 e Å^−^^3^
229 parameters	Extinction correction: *SHELXL2014* (Sheldrick, 2015), Fc^*^=kFc[1+0.001xFc^2^λ^3^/sin(2θ)]^-1/4^
0 restraints	Extinction coefficient: 0.00048 (6)

### (II) Methyl 5-bromo-2-[(2-chloro-6-methylquinolin-3-yl)methoxy]benzoate Special details

**Table:**

Geometry. All e.s.d.'s (except the e.s.d. in the dihedral angle between two l.s. planes) are estimated using the full covariance matrix. The cell e.s.d.'s are taken into account individually in the estimation of e.s.d.'s in distances, angles and torsion angles; correlations between e.s.d.'s in cell parameters are only used when they are defined by crystal symmetry. An approximate (isotropic) treatment of cell e.s.d.'s is used for estimating e.s.d.'s involving l.s. planes.

### (II) Methyl 5-bromo-2-[(2-chloro-6-methylquinolin-3-yl)methoxy]benzoate Fractional atomic coordinates and isotropic or equivalent isotropic displacement parameters (Å^2^)

**Table:**

	*x*	*y*	*z*	*U*_iso_*/*U*_eq_	
N1	0.42983 (12)	0.87307 (15)	0.45790 (10)	0.0272 (4)	
C2	0.42690 (13)	0.78272 (18)	0.42079 (11)	0.0249 (4)	
Cl2	0.52936 (3)	0.72811 (5)	0.39507 (3)	0.03655 (16)	
C3	0.35033 (14)	0.72343 (17)	0.40030 (10)	0.0226 (4)	
C4	0.27189 (13)	0.76626 (18)	0.42260 (11)	0.0224 (4)	
H4	0.2185	0.7297	0.4106	0.027*	
C4A	0.26987 (13)	0.86525 (18)	0.46363 (11)	0.0219 (4)	
C5	0.19142 (14)	0.91446 (18)	0.48888 (11)	0.0253 (4)	
H5	0.1366	0.8805	0.4780	0.030*	
C6	0.19242 (15)	1.00976 (18)	0.52857 (11)	0.0267 (4)	
C7	0.27500 (15)	1.05991 (19)	0.54421 (12)	0.0302 (5)	
H7	0.2765	1.1261	0.5716	0.036*	
C8	0.35220 (15)	1.01485 (19)	0.52062 (12)	0.0299 (5)	
H8	0.4066	1.0499	0.5316	0.036*	
C8A	0.35123 (14)	0.91644 (17)	0.48007 (11)	0.0242 (4)	
C37	0.35875 (13)	0.61865 (18)	0.35765 (11)	0.0243 (4)	
H37A	0.3916	0.6338	0.3137	0.029*	
H37B	0.3909	0.5610	0.3846	0.029*	
O31	0.27182 (9)	0.58119 (13)	0.34187 (8)	0.0254 (3)	
C31	0.26370 (13)	0.48636 (18)	0.30374 (11)	0.0224 (4)	
C32	0.17885 (14)	0.45623 (18)	0.28094 (11)	0.0236 (4)	
C33	0.16860 (15)	0.36086 (18)	0.23979 (11)	0.0267 (4)	
H33	0.1116	0.3399	0.2240	0.032*	
C34	0.24049 (16)	0.29669 (19)	0.22183 (11)	0.0271 (5)	
Br34	0.22547 (2)	0.17010 (2)	0.16308 (2)	0.03596 (12)	
C35	0.32387 (16)	0.32597 (18)	0.24421 (12)	0.0297 (5)	
H35	0.3731	0.2813	0.2317	0.036*	
C36	0.33537 (14)	0.42003 (18)	0.28463 (11)	0.0273 (5)	
H36	0.3928	0.4401	0.2997	0.033*	
C38	0.09631 (14)	0.5209 (2)	0.29458 (12)	0.0309 (5)	
O38	0.03146 (13)	0.5097 (2)	0.25963 (13)	0.0739 (8)	
O39	0.10112 (11)	0.59054 (16)	0.34798 (9)	0.0380 (4)	
C39	0.02126 (19)	0.6494 (3)	0.36541 (18)	0.0522 (7)	
H39A	0.0089	0.7052	0.3291	0.078*	
H39B	0.0283	0.6866	0.4109	0.078*	
H39C	−0.0277	0.5963	0.3680	0.078*	
C61	0.10926 (16)	1.0618 (2)	0.55704 (13)	0.0365 (5)	
H61A	0.0590	1.0382	0.5284	0.055*	
H61B	0.1145	1.1432	0.5555	0.055*	
H61C	0.1003	1.0376	0.6057	0.055*	

### (II) Methyl 5-bromo-2-[(2-chloro-6-methylquinolin-3-yl)methoxy]benzoate Atomic displacement parameters (Å^2^)

**Table:**

	*U*^11^	*U*^22^	*U*^33^	*U*^12^	*U*^13^	*U*^23^
N1	0.0241 (9)	0.0257 (9)	0.0319 (10)	−0.0054 (7)	−0.0027 (7)	−0.0008 (8)
C2	0.0193 (10)	0.0264 (11)	0.0291 (11)	−0.0017 (8)	0.0002 (8)	0.0026 (8)
Cl2	0.0207 (3)	0.0373 (3)	0.0516 (4)	−0.0023 (2)	0.0022 (2)	−0.0088 (3)
C3	0.0236 (10)	0.0229 (10)	0.0214 (10)	−0.0037 (8)	−0.0022 (8)	0.0021 (8)
C4	0.0202 (10)	0.0249 (10)	0.0222 (10)	−0.0041 (8)	−0.0023 (7)	0.0023 (8)
C4A	0.0236 (10)	0.0226 (10)	0.0196 (10)	−0.0023 (8)	−0.0023 (7)	0.0033 (8)
C5	0.0248 (10)	0.0288 (11)	0.0224 (10)	−0.0030 (9)	−0.0021 (8)	0.0012 (8)
C6	0.0309 (11)	0.0263 (11)	0.0228 (10)	0.0025 (9)	−0.0017 (8)	0.0019 (8)
C7	0.0397 (13)	0.0231 (10)	0.0276 (11)	−0.0022 (9)	−0.0023 (9)	−0.0035 (9)
C8	0.0308 (11)	0.0263 (11)	0.0325 (11)	−0.0059 (9)	−0.0045 (9)	−0.0022 (9)
C8A	0.0262 (10)	0.0227 (10)	0.0238 (10)	−0.0040 (8)	−0.0038 (8)	0.0014 (8)
C37	0.0199 (10)	0.0245 (10)	0.0285 (11)	−0.0029 (8)	−0.0007 (8)	−0.0021 (9)
O31	0.0198 (7)	0.0252 (8)	0.0312 (8)	−0.0013 (6)	−0.0016 (5)	−0.0079 (6)
C31	0.0241 (10)	0.0226 (10)	0.0204 (10)	−0.0015 (8)	0.0002 (7)	0.0013 (8)
C32	0.0230 (10)	0.0237 (10)	0.0242 (10)	−0.0026 (8)	−0.0009 (8)	0.0037 (8)
C33	0.0283 (11)	0.0279 (11)	0.0238 (10)	−0.0068 (9)	−0.0032 (8)	0.0032 (9)
C34	0.0407 (12)	0.0212 (10)	0.0195 (10)	−0.0054 (9)	−0.0007 (9)	−0.0012 (8)
Br34	0.0551 (2)	0.02513 (16)	0.02764 (17)	−0.00542 (10)	−0.00230 (10)	−0.00488 (9)
C35	0.0322 (11)	0.0286 (12)	0.0283 (11)	0.0027 (9)	0.0045 (9)	−0.0016 (9)
C36	0.0225 (10)	0.0300 (12)	0.0294 (11)	−0.0023 (9)	0.0004 (8)	−0.0022 (9)
C38	0.0225 (10)	0.0334 (12)	0.0369 (12)	−0.0003 (9)	−0.0027 (9)	−0.0007 (10)
O38	0.0309 (10)	0.100 (2)	0.0905 (17)	0.0164 (11)	−0.0257 (11)	−0.0441 (16)
O39	0.0232 (8)	0.0460 (11)	0.0446 (10)	0.0068 (7)	0.0006 (7)	−0.0115 (8)
C39	0.0298 (13)	0.0636 (19)	0.0632 (19)	0.0151 (13)	0.0099 (13)	−0.0070 (16)
C61	0.0372 (13)	0.0366 (13)	0.0357 (13)	0.0042 (10)	0.0022 (10)	−0.0082 (10)

### (II) Methyl 5-bromo-2-[(2-chloro-6-methylquinolin-3-yl)methoxy]benzoate Geometric parameters (Å, º)

**Table:**

N1—C2	1.294 (3)	O31—C31	1.354 (3)
N1—C8A	1.369 (3)	C31—C36	1.396 (3)
C2—C3	1.418 (3)	C31—C32	1.407 (3)
C2—Cl2	1.758 (2)	C32—C33	1.394 (3)
C3—C4	1.365 (3)	C32—C38	1.497 (3)
C3—C37	1.501 (3)	C33—C34	1.379 (3)
C4—C4A	1.421 (3)	C33—H33	0.9500
C4—H4	0.9500	C34—C35	1.382 (3)
C4A—C5	1.414 (3)	C34—Br34	1.899 (2)
C4A—C8A	1.415 (3)	C35—C36	1.376 (3)
C5—C6	1.370 (3)	C35—H35	0.9500
C5—H5	0.9500	C36—H36	0.9500
C6—C7	1.423 (3)	C38—O38	1.196 (3)
C6—C61	1.509 (3)	C38—O39	1.317 (3)
C7—C8	1.367 (3)	O39—C39	1.442 (3)
C7—H7	0.9500	C39—H39A	0.9800
C8—C8A	1.410 (3)	C39—H39B	0.9800
C8—H8	0.9500	C39—H39C	0.9800
C37—O31	1.427 (2)	C61—H61A	0.9800
C37—H37A	0.9900	C61—H61B	0.9800
C37—H37B	0.9900	C61—H61C	0.9800
			
C2—N1—C8A	117.13 (18)	O31—C31—C36	123.12 (19)
N1—C2—C3	126.73 (19)	O31—C31—C32	117.66 (18)
N1—C2—Cl2	115.67 (15)	C36—C31—C32	119.2 (2)
C3—C2—Cl2	117.59 (16)	C33—C32—C31	119.1 (2)
C4—C3—C2	116.24 (19)	C33—C32—C38	115.38 (19)
C4—C3—C37	123.86 (18)	C31—C32—C38	125.50 (19)
C2—C3—C37	119.89 (19)	C34—C33—C32	120.5 (2)
C3—C4—C4A	120.25 (18)	C34—C33—H33	119.7
C3—C4—H4	119.9	C32—C33—H33	119.7
C4A—C4—H4	119.9	C33—C34—C35	120.5 (2)
C5—C4A—C8A	118.7 (2)	C33—C34—Br34	119.77 (17)
C5—C4A—C4	123.60 (18)	C35—C34—Br34	119.65 (18)
C8A—C4A—C4	117.70 (18)	C36—C35—C34	119.8 (2)
C6—C5—C4A	121.7 (2)	C36—C35—H35	120.1
C6—C5—H5	119.1	C34—C35—H35	120.1
C4A—C5—H5	119.1	C35—C36—C31	120.9 (2)
C5—C6—C7	118.5 (2)	C35—C36—H36	119.6
C5—C6—C61	122.2 (2)	C31—C36—H36	119.6
C7—C6—C61	119.2 (2)	O38—C38—O39	123.1 (2)
C8—C7—C6	121.4 (2)	O38—C38—C32	122.3 (2)
C8—C7—H7	119.3	O39—C38—C32	114.56 (18)
C6—C7—H7	119.3	C38—O39—C39	116.1 (2)
C7—C8—C8A	120.1 (2)	O39—C39—H39A	109.5
C7—C8—H8	120.0	O39—C39—H39B	109.5
C8A—C8—H8	120.0	H39A—C39—H39B	109.5
N1—C8A—C8	118.51 (19)	O39—C39—H39C	109.5
N1—C8A—C4A	121.94 (19)	H39A—C39—H39C	109.5
C8—C8A—C4A	119.5 (2)	H39B—C39—H39C	109.5
O31—C37—C3	107.35 (16)	C6—C61—H61A	109.5
O31—C37—H37A	110.2	C6—C61—H61B	109.5
C3—C37—H37A	110.2	H61A—C61—H61B	109.5
O31—C37—H37B	110.2	C6—C61—H61C	109.5
C3—C37—H37B	110.2	H61A—C61—H61C	109.5
H37A—C37—H37B	108.5	H61B—C61—H61C	109.5
C31—O31—C37	117.47 (16)		
			
C8A—N1—C2—C3	0.2 (3)	C4—C3—C37—O31	4.3 (3)
C8A—N1—C2—Cl2	−178.63 (15)	C2—C3—C37—O31	−176.93 (18)
N1—C2—C3—C4	−0.4 (3)	C3—C37—O31—C31	−179.57 (17)
Cl2—C2—C3—C4	178.35 (16)	C37—O31—C31—C36	5.4 (3)
N1—C2—C3—C37	−179.3 (2)	C37—O31—C31—C32	−172.62 (18)
Cl2—C2—C3—C37	−0.5 (3)	O31—C31—C32—C33	178.09 (18)
C2—C3—C4—C4A	0.3 (3)	C36—C31—C32—C33	0.0 (3)
C37—C3—C4—C4A	179.16 (19)	O31—C31—C32—C38	0.7 (3)
C3—C4—C4A—C5	−179.75 (19)	C36—C31—C32—C38	−177.4 (2)
C3—C4—C4A—C8A	0.0 (3)	C31—C32—C33—C34	0.2 (3)
C8A—C4A—C5—C6	0.0 (3)	C38—C32—C33—C34	177.8 (2)
C4—C4A—C5—C6	179.7 (2)	C32—C33—C34—C35	−0.1 (3)
C4A—C5—C6—C7	0.2 (3)	C32—C33—C34—Br34	−177.84 (16)
C4A—C5—C6—C61	−178.7 (2)	C33—C34—C35—C36	−0.1 (3)
C5—C6—C7—C8	−0.1 (3)	Br34—C34—C35—C36	177.58 (17)
C61—C6—C7—C8	178.9 (2)	C34—C35—C36—C31	0.3 (3)
C6—C7—C8—C8A	−0.2 (4)	O31—C31—C36—C35	−178.2 (2)
C2—N1—C8A—C8	180.0 (2)	C32—C31—C36—C35	−0.2 (3)
C2—N1—C8A—C4A	0.2 (3)	C33—C32—C38—O38	−18.0 (4)
C7—C8—C8A—N1	−179.4 (2)	C31—C32—C38—O38	159.5 (3)
C7—C8—C8A—C4A	0.4 (3)	C33—C32—C38—O39	161.8 (2)
C5—C4A—C8A—N1	179.49 (19)	C31—C32—C38—O39	−20.7 (3)
C4—C4A—C8A—N1	−0.3 (3)	O38—C38—O39—C39	3.5 (4)
C5—C4A—C8A—C8	−0.3 (3)	C32—C38—O39—C39	−176.4 (2)
C4—C4A—C8A—C8	179.95 (19)		

### (II) Methyl 5-bromo-2-[(2-chloro-6-methylquinolin-3-yl)methoxy]benzoate Hydrogen-bond geometry (Å, º)

**Table:**

*D*—H···*A*	*D*—H	H···*A*	*D*···*A*	*D*—H···*A*
C36—H36···O38^i^	0.95	2.53	3.277 (3)	136

Symmetry code: (i) *x*+1/2, *y*, −*z*+1/2.

### (III) Methyl 2-[(2-chloro-6-methylquinolin-3-yl)methoxy]benzoate Crystal data

**Table:**

C_19_H_16_ClNO_3_	*D*_x_ = 1.386 Mg m^−^^3^
*M**_r_* = 341.78	Cu *K*α radiation, λ = 1.54184 Å
Orthorhombic, *P*2_1_2_1_2_1_	Cell parameters from 12841 reflections
*a* = 13.5860 (3) Å	θ = 3.6–72.6°
*b* = 15.5857 (2) Å	µ = 2.21 mm^−^^1^
*c* = 30.9389 (5) Å	*T* = 173 K
*V* = 6551.2 (2) Å^3^	Block, colourless
*Z* = 16	0.48 × 0.26 × 0.14 mm
*F*(000) = 2848	

### (III) Methyl 2-[(2-chloro-6-methylquinolin-3-yl)methoxy]benzoate Data collection

**Table:**

Agilent Eos Gemini diffractometer	11257 reflections with *I* > 2σ(*I*)
Radiation source: Enhance (Cu) X-ray Source	*R*_int_ = 0.048
ω scans	θ_max_ = 72.6°, θ_min_ = 3.6°
Absorption correction: multi-scan (*SADABS*; Sheldrick, 2003)	*h* = −16→15
*T*_min_ = 0.472, *T*_max_ = 0.734	*k* = −19→15
45901 measured reflections	*l* = −38→30
12840 independent reflections	

### (III) Methyl 2-[(2-chloro-6-methylquinolin-3-yl)methoxy]benzoate Refinement

**Table:**

Refinement on *F*^2^	Hydrogen site location: inferred from neighbouring sites
Least-squares matrix: full	H-atom parameters constrained
*R*[*F*^2^ > 2σ(*F*^2^)] = 0.046	*w* = 1/[σ^2^(*F*_o_^2^) + (0.0759*P*)^2^ + 0.9452*P*] where *P* = (*F*_o_^2^ + 2*F*_c_^2^)/3
*wR*(*F*^2^) = 0.129	(Δ/σ)_max_ = 0.001
*S* = 1.04	Δρ_max_ = 0.42 e Å^−^^3^
12840 reflections	Δρ_min_ = −0.31 e Å^−^^3^
874 parameters	Absolute structure: Refined as an inversion twin.
0 restraints	Absolute structure parameter: 0.152 (16)

### (III) Methyl 2-[(2-chloro-6-methylquinolin-3-yl)methoxy]benzoate Special details

**Table:**

Geometry. All e.s.d.'s (except the e.s.d. in the dihedral angle between two l.s. planes) are estimated using the full covariance matrix. The cell e.s.d.'s are taken into account individually in the estimation of e.s.d.'s in distances, angles and torsion angles; correlations between e.s.d.'s in cell parameters are only used when they are defined by crystal symmetry. An approximate (isotropic) treatment of cell e.s.d.'s is used for estimating e.s.d.'s involving l.s. planes.
Refinement. Refined as a 2-component inversion twin.

### (III) Methyl 2-[(2-chloro-6-methylquinolin-3-yl)methoxy]benzoate Fractional atomic coordinates and isotropic or equivalent isotropic displacement parameters (Å^2^)

**Table:**

	*x*	*y*	*z*	*U*_iso_*/*U*_eq_	
N11	0.0311 (2)	0.26008 (18)	0.44748 (8)	0.0301 (6)	
C12	0.0259 (3)	0.2694 (2)	0.40606 (10)	0.0293 (7)	
Cl12	0.02044 (8)	0.17391 (5)	0.37626 (3)	0.0430 (2)	
C13	0.0238 (2)	0.3474 (2)	0.38287 (10)	0.0268 (6)	
C14	0.0259 (2)	0.4211 (2)	0.40728 (10)	0.0282 (7)	
H14	0.0256	0.4756	0.3935	0.034*	
C14A	0.0287 (2)	0.4159 (2)	0.45268 (11)	0.0282 (7)	
C15	0.0243 (3)	0.4892 (2)	0.47997 (12)	0.0332 (7)	
H15	0.0226	0.5447	0.4674	0.040*	
C16	0.0225 (3)	0.4814 (3)	0.52403 (12)	0.0363 (8)	
C17	0.0282 (3)	0.3981 (3)	0.54256 (11)	0.0382 (8)	
H17	0.0286	0.3923	0.5731	0.046*	
C18	0.0332 (3)	0.3261 (2)	0.51731 (11)	0.0348 (7)	
H18	0.0371	0.2710	0.5304	0.042*	
C18A	0.0325 (2)	0.3337 (2)	0.47186 (11)	0.0290 (7)	
C137	0.0188 (3)	0.3476 (2)	0.33422 (10)	0.0302 (7)	
H13A	0.0764	0.3171	0.3220	0.036*	
H13B	−0.0418	0.3184	0.3243	0.036*	
O131	0.0187 (2)	0.43457 (15)	0.32062 (7)	0.0346 (5)	
C131	0.0188 (3)	0.4508 (2)	0.27728 (10)	0.0277 (7)	
C132	0.0243 (3)	0.5371 (2)	0.26405 (10)	0.0284 (7)	
C133	0.0259 (3)	0.5540 (2)	0.21978 (11)	0.0338 (8)	
H133	0.0303	0.6119	0.2103	0.041*	
C134	0.0215 (3)	0.4895 (3)	0.18938 (11)	0.0404 (9)	
H134	0.0230	0.5027	0.1594	0.048*	
C135	0.0147 (3)	0.4051 (3)	0.20307 (12)	0.0391 (8)	
H135	0.0107	0.3602	0.1824	0.047*	
C136	0.0138 (3)	0.3858 (2)	0.24656 (11)	0.0349 (8)	
H136	0.0097	0.3276	0.2556	0.042*	
C138	0.0272 (3)	0.6138 (2)	0.29250 (11)	0.0318 (7)	
O138	0.0287 (3)	0.68600 (18)	0.27853 (9)	0.0590 (8)	
O139	0.0286 (2)	0.59794 (16)	0.33477 (8)	0.0362 (6)	
C139	0.0327 (3)	0.6724 (3)	0.36214 (12)	0.0412 (9)	
H19A	0.0351	0.6543	0.3925	0.062*	
H19B	−0.0259	0.7077	0.3574	0.062*	
H19C	0.0917	0.7058	0.3553	0.062*	
C161	0.0161 (4)	0.5585 (3)	0.55315 (13)	0.0489 (10)	
H16A	0.0696	0.5566	0.5743	0.073*	
H16B	−0.0473	0.5583	0.5683	0.073*	
H16C	0.0217	0.6109	0.5358	0.073*	
N21	0.2866 (2)	0.67705 (19)	0.53534 (10)	0.0345 (6)	
C22	0.2884 (3)	0.6675 (2)	0.49411 (11)	0.0311 (7)	
Cl22	0.30603 (8)	0.76266 (6)	0.46423 (3)	0.0463 (2)	
C23	0.2803 (2)	0.5902 (2)	0.47028 (11)	0.0297 (7)	
C24	0.2724 (3)	0.5170 (2)	0.49451 (11)	0.0312 (7)	
H24	0.2675	0.4628	0.4806	0.037*	
C24A	0.2716 (3)	0.5217 (2)	0.54036 (11)	0.0302 (7)	
C25	0.2632 (3)	0.4488 (2)	0.56743 (12)	0.0359 (8)	
H25	0.2604	0.3933	0.5548	0.043*	
C26	0.2590 (3)	0.4568 (3)	0.61136 (13)	0.0406 (9)	
C27	0.2650 (3)	0.5396 (3)	0.62980 (13)	0.0434 (9)	
H27	0.2622	0.5453	0.6603	0.052*	
C28	0.2746 (3)	0.6112 (3)	0.60521 (12)	0.0388 (8)	
H28	0.2794	0.6660	0.6186	0.047*	
C28A	0.2774 (3)	0.6041 (2)	0.55952 (12)	0.0332 (7)	
C237	0.2795 (3)	0.5903 (2)	0.42186 (11)	0.0320 (7)	
H23A	0.2233	0.6245	0.4110	0.038*	
H23B	0.3411	0.6158	0.4106	0.038*	
O231	0.2709 (2)	0.50396 (16)	0.40813 (8)	0.0375 (6)	
C231	0.2748 (3)	0.4863 (2)	0.36514 (11)	0.0301 (7)	
C232	0.2748 (3)	0.3993 (2)	0.35269 (11)	0.0294 (7)	
C233	0.2777 (3)	0.3806 (2)	0.30865 (12)	0.0367 (8)	
H233	0.2774	0.3223	0.2998	0.044*	
C234	0.2811 (3)	0.4434 (3)	0.27777 (12)	0.0392 (9)	
H234	0.2836	0.4286	0.2480	0.047*	
C235	0.2810 (3)	0.5288 (3)	0.29027 (12)	0.0383 (8)	
H235	0.2827	0.5727	0.2690	0.046*	
C236	0.2782 (3)	0.5503 (2)	0.33367 (12)	0.0330 (7)	
H236	0.2787	0.6090	0.3421	0.040*	
C238	0.2727 (3)	0.3238 (2)	0.38217 (12)	0.0331 (8)	
O238	0.2553 (3)	0.25274 (18)	0.36991 (10)	0.0546 (8)	
O239	0.2936 (2)	0.34248 (17)	0.42314 (8)	0.0417 (6)	
C239	0.2981 (3)	0.2700 (3)	0.45206 (13)	0.0485 (10)	
H29A	0.3414	0.2260	0.4398	0.073*	
H29B	0.2319	0.2462	0.4559	0.073*	
H29C	0.3241	0.2885	0.4801	0.073*	
C261	0.2477 (4)	0.3792 (3)	0.64007 (14)	0.0528 (11)	
H26A	0.3075	0.3716	0.6574	0.079*	
H26B	0.2372	0.3281	0.6222	0.079*	
H26C	0.1912	0.3873	0.6593	0.079*	
N31	0.5271 (2)	0.39181 (18)	0.44626 (9)	0.0282 (6)	
C32	0.5293 (2)	0.3996 (2)	0.40484 (10)	0.0272 (7)	
Cl32	0.53360 (8)	0.30319 (5)	0.37552 (3)	0.0403 (2)	
C33	0.5300 (2)	0.4771 (2)	0.38069 (10)	0.0253 (6)	
C34	0.5317 (2)	0.5512 (2)	0.40443 (10)	0.0272 (7)	
H34	0.5335	0.6052	0.3902	0.033*	
C34A	0.5309 (3)	0.5474 (2)	0.44993 (11)	0.0277 (7)	
C35	0.5330 (3)	0.6216 (2)	0.47646 (12)	0.0345 (8)	
H35	0.5362	0.6766	0.4633	0.041*	
C36	0.5305 (3)	0.6157 (3)	0.52091 (12)	0.0387 (8)	
C37	0.5264 (3)	0.5331 (3)	0.53988 (11)	0.0401 (8)	
H37	0.5248	0.5285	0.5705	0.048*	
C38	0.5248 (3)	0.4602 (3)	0.51574 (11)	0.0356 (8)	
H38	0.5219	0.4057	0.5295	0.043*	
C38A	0.5275 (3)	0.4658 (2)	0.46987 (10)	0.0279 (7)	
C337	0.5297 (3)	0.4760 (2)	0.33200 (10)	0.0261 (6)	
H33A	0.5895	0.4469	0.3210	0.031*	
H33B	0.4711	0.4450	0.3211	0.031*	
O331	0.5278 (2)	0.56258 (14)	0.31813 (7)	0.0317 (5)	
C331	0.5256 (2)	0.5789 (2)	0.27513 (10)	0.0265 (6)	
C332	0.5200 (3)	0.6657 (2)	0.26216 (10)	0.0299 (7)	
C333	0.5204 (3)	0.6837 (2)	0.21809 (11)	0.0368 (8)	
H333	0.5178	0.7418	0.2089	0.044*	
C334	0.5244 (3)	0.6196 (3)	0.18732 (11)	0.0417 (9)	
H334	0.5254	0.6336	0.1574	0.050*	
C335	0.5269 (3)	0.5350 (2)	0.20050 (11)	0.0372 (8)	
H335	0.5282	0.4905	0.1795	0.045*	
C336	0.5277 (3)	0.5144 (2)	0.24398 (11)	0.0316 (7)	
H336	0.5297	0.4560	0.2527	0.038*	
C338	0.5135 (3)	0.7414 (2)	0.29139 (11)	0.0339 (7)	
O338	0.4943 (3)	0.81240 (18)	0.27849 (10)	0.0545 (8)	
O339	0.5325 (2)	0.72473 (16)	0.33255 (8)	0.0430 (6)	
C339	0.5292 (4)	0.7983 (3)	0.36114 (13)	0.0584 (12)	
H39A	0.5762	0.8417	0.3511	0.088*	
H39B	0.5466	0.7803	0.3905	0.088*	
H39C	0.4627	0.8226	0.3611	0.088*	
C361	0.5325 (4)	0.6949 (3)	0.54871 (13)	0.0520 (11)	
H36A	0.5506	0.7446	0.5310	0.078*	
H36B	0.5810	0.6875	0.5719	0.078*	
H36C	0.4673	0.7043	0.5614	0.078*	
N41	0.7784 (2)	0.67216 (19)	0.35785 (9)	0.0312 (6)	
C42	0.7768 (2)	0.6643 (2)	0.31639 (11)	0.0276 (7)	
Cl42	0.77365 (8)	0.76085 (5)	0.28715 (3)	0.0395 (2)	
C43	0.7776 (2)	0.5869 (2)	0.29224 (11)	0.0265 (7)	
C44	0.7803 (2)	0.5131 (2)	0.31580 (11)	0.0283 (7)	
H44	0.7824	0.4593	0.3014	0.034*	
C44A	0.7802 (2)	0.5158 (2)	0.36136 (11)	0.0278 (7)	
C45	0.7793 (3)	0.4418 (2)	0.38798 (12)	0.0338 (8)	
H45	0.7787	0.3867	0.3749	0.041*	
C46	0.7792 (3)	0.4481 (3)	0.43207 (13)	0.0371 (9)	
C47	0.7801 (3)	0.5302 (3)	0.45138 (12)	0.0393 (9)	
H47	0.7802	0.5347	0.4820	0.047*	
C48	0.7811 (3)	0.6034 (3)	0.42712 (12)	0.0371 (8)	
H48	0.7825	0.6578	0.4409	0.045*	
C48A	0.7799 (2)	0.5982 (2)	0.38153 (10)	0.0284 (7)	
C437	0.7759 (3)	0.5886 (2)	0.24373 (10)	0.0287 (7)	
H43A	0.7172	0.6200	0.2333	0.034*	
H43B	0.8355	0.6176	0.2325	0.034*	
O431	0.77309 (19)	0.50187 (15)	0.22965 (8)	0.0334 (5)	
C431	0.7656 (3)	0.4859 (2)	0.18670 (11)	0.0294 (7)	
C432	0.7540 (2)	0.4005 (2)	0.17336 (11)	0.0314 (7)	
C433	0.7493 (3)	0.3835 (2)	0.12886 (12)	0.0388 (8)	
H433	0.7435	0.3257	0.1194	0.047*	
C434	0.7528 (3)	0.4479 (3)	0.09854 (12)	0.0452 (10)	
H434	0.7488	0.4348	0.0686	0.054*	
C435	0.7621 (3)	0.5321 (3)	0.11217 (12)	0.0420 (9)	
H435	0.7647	0.5770	0.0915	0.050*	
C436	0.7678 (3)	0.5512 (2)	0.15565 (12)	0.0353 (8)	
H436	0.7732	0.6093	0.1646	0.042*	
C438	0.7472 (3)	0.3232 (2)	0.20179 (12)	0.0347 (8)	
O438	0.7358 (3)	0.25227 (18)	0.18788 (10)	0.0560 (8)	
O439	0.7541 (2)	0.33994 (16)	0.24416 (8)	0.0404 (6)	
C439	0.7475 (4)	0.2656 (3)	0.27155 (13)	0.0501 (10)	
H49A	0.7496	0.2835	0.3019	0.075*	
H49B	0.8030	0.2272	0.2656	0.075*	
H49C	0.6856	0.2355	0.2659	0.075*	
C461	0.7757 (3)	0.3687 (3)	0.46052 (14)	0.0498 (11)	
H46A	0.7075	0.3576	0.4693	0.075*	
H46B	0.8163	0.3780	0.4863	0.075*	
H46C	0.8009	0.3193	0.4444	0.075*	

### (III) Methyl 2-[(2-chloro-6-methylquinolin-3-yl)methoxy]benzoate Atomic displacement parameters (Å^2^)

**Table:**

	*U*^11^	*U*^22^	*U*^33^	*U*^12^	*U*^13^	*U*^23^
N11	0.0385 (15)	0.0228 (13)	0.0289 (13)	0.0008 (12)	−0.0018 (12)	0.0024 (11)
C12	0.0348 (17)	0.0208 (15)	0.0324 (16)	0.0002 (14)	−0.0029 (14)	−0.0009 (13)
Cl12	0.0731 (6)	0.0208 (4)	0.0351 (4)	−0.0022 (4)	−0.0056 (4)	−0.0023 (3)
C13	0.0278 (15)	0.0240 (16)	0.0286 (16)	0.0016 (13)	−0.0003 (13)	0.0023 (12)
C14	0.0306 (16)	0.0225 (16)	0.0315 (16)	−0.0009 (13)	−0.0002 (14)	0.0040 (13)
C14A	0.0254 (15)	0.0247 (16)	0.0345 (17)	−0.0018 (13)	−0.0005 (13)	−0.0019 (13)
C15	0.0321 (17)	0.0271 (17)	0.0404 (19)	0.0011 (14)	0.0004 (15)	−0.0037 (14)
C16	0.0306 (17)	0.040 (2)	0.039 (2)	−0.0001 (16)	−0.0027 (15)	−0.0103 (16)
C17	0.0402 (19)	0.048 (2)	0.0259 (16)	−0.0039 (18)	−0.0017 (15)	−0.0029 (15)
C18	0.0391 (19)	0.0345 (18)	0.0309 (17)	−0.0009 (16)	−0.0023 (15)	0.0030 (15)
C18A	0.0266 (16)	0.0276 (16)	0.0329 (17)	−0.0010 (13)	−0.0019 (13)	0.0001 (14)
C137	0.0413 (18)	0.0206 (15)	0.0287 (16)	0.0009 (14)	−0.0029 (14)	0.0027 (13)
O131	0.0548 (15)	0.0209 (11)	0.0282 (12)	0.0041 (11)	−0.0016 (11)	0.0038 (9)
C131	0.0296 (16)	0.0256 (16)	0.0279 (16)	0.0039 (13)	0.0008 (13)	0.0048 (13)
C132	0.0284 (16)	0.0270 (17)	0.0298 (17)	0.0045 (14)	0.0011 (13)	0.0042 (13)
C133	0.0373 (18)	0.0323 (18)	0.0316 (18)	0.0075 (15)	0.0034 (15)	0.0107 (14)
C134	0.049 (2)	0.046 (2)	0.0266 (17)	0.0104 (19)	0.0010 (16)	0.0043 (16)
C135	0.049 (2)	0.0355 (19)	0.0326 (18)	0.0083 (17)	−0.0029 (16)	−0.0056 (15)
C136	0.044 (2)	0.0251 (17)	0.0359 (18)	0.0066 (15)	0.0004 (15)	−0.0002 (14)
C138	0.0357 (18)	0.0251 (17)	0.0345 (17)	0.0045 (14)	−0.0010 (14)	0.0045 (14)
O138	0.108 (3)	0.0250 (14)	0.0440 (16)	0.0015 (16)	−0.0068 (17)	0.0038 (12)
O139	0.0511 (15)	0.0257 (12)	0.0318 (12)	−0.0007 (11)	−0.0024 (11)	−0.0004 (10)
C139	0.053 (2)	0.0323 (19)	0.0382 (19)	−0.0024 (18)	−0.0049 (17)	−0.0082 (16)
C161	0.054 (2)	0.049 (2)	0.044 (2)	0.003 (2)	0.0007 (19)	−0.0149 (19)
N21	0.0440 (17)	0.0262 (15)	0.0332 (15)	0.0035 (13)	−0.0030 (13)	−0.0036 (12)
C22	0.0377 (18)	0.0238 (16)	0.0318 (17)	0.0025 (14)	−0.0026 (13)	0.0006 (14)
Cl22	0.0791 (7)	0.0235 (4)	0.0363 (4)	0.0029 (4)	−0.0059 (4)	0.0019 (4)
C23	0.0307 (16)	0.0286 (17)	0.0297 (17)	0.0046 (14)	−0.0027 (13)	−0.0045 (14)
C24	0.0332 (17)	0.0237 (16)	0.0366 (19)	0.0004 (14)	−0.0022 (14)	−0.0045 (14)
C24A	0.0281 (16)	0.0295 (17)	0.0330 (18)	−0.0005 (14)	−0.0010 (14)	−0.0025 (14)
C25	0.0371 (19)	0.0314 (18)	0.039 (2)	−0.0024 (15)	−0.0011 (15)	0.0001 (15)
C26	0.040 (2)	0.045 (2)	0.037 (2)	−0.0038 (17)	−0.0008 (16)	0.0092 (17)
C27	0.046 (2)	0.054 (2)	0.0295 (18)	−0.0019 (19)	−0.0019 (16)	−0.0007 (17)
C28	0.048 (2)	0.039 (2)	0.0296 (18)	0.0000 (17)	−0.0002 (16)	−0.0060 (15)
C28A	0.0322 (17)	0.0314 (18)	0.0359 (18)	0.0022 (15)	−0.0013 (14)	−0.0021 (15)
C237	0.0403 (19)	0.0230 (16)	0.0328 (18)	0.0032 (14)	−0.0021 (14)	−0.0036 (14)
O231	0.0597 (16)	0.0244 (12)	0.0285 (13)	−0.0032 (11)	0.0045 (11)	−0.0034 (10)
C231	0.0297 (16)	0.0290 (17)	0.0316 (18)	−0.0023 (14)	−0.0020 (13)	−0.0035 (13)
C232	0.0286 (16)	0.0276 (17)	0.0321 (17)	−0.0028 (14)	−0.0032 (13)	−0.0032 (14)
C233	0.042 (2)	0.0306 (18)	0.0378 (19)	−0.0064 (16)	−0.0020 (16)	−0.0099 (15)
C234	0.048 (2)	0.043 (2)	0.0268 (18)	−0.0062 (18)	−0.0065 (15)	−0.0023 (16)
C235	0.041 (2)	0.040 (2)	0.0338 (19)	0.0003 (16)	−0.0053 (15)	0.0082 (16)
C236	0.0348 (18)	0.0275 (17)	0.0368 (19)	0.0012 (14)	−0.0039 (15)	0.0011 (14)
C238	0.0301 (16)	0.0297 (18)	0.039 (2)	−0.0025 (14)	0.0013 (14)	−0.0053 (15)
O238	0.088 (2)	0.0271 (14)	0.0490 (17)	−0.0156 (14)	−0.0052 (15)	−0.0007 (13)
O239	0.0632 (18)	0.0287 (13)	0.0330 (13)	0.0012 (12)	−0.0076 (12)	0.0028 (11)
C239	0.065 (3)	0.036 (2)	0.045 (2)	0.0049 (19)	−0.0081 (19)	0.0128 (18)
C261	0.061 (3)	0.049 (3)	0.048 (2)	−0.005 (2)	0.001 (2)	0.015 (2)
N31	0.0335 (14)	0.0237 (14)	0.0274 (13)	−0.0006 (12)	−0.0016 (11)	0.0024 (11)
C32	0.0324 (16)	0.0188 (15)	0.0302 (16)	−0.0016 (13)	−0.0026 (14)	−0.0014 (13)
Cl32	0.0723 (6)	0.0187 (4)	0.0299 (4)	−0.0005 (4)	−0.0027 (4)	−0.0015 (3)
C33	0.0249 (14)	0.0211 (15)	0.0300 (16)	0.0011 (12)	0.0012 (13)	0.0023 (13)
C34	0.0304 (16)	0.0203 (15)	0.0308 (16)	0.0017 (13)	0.0005 (14)	0.0026 (12)
C34A	0.0262 (16)	0.0268 (17)	0.0300 (17)	0.0016 (13)	−0.0013 (13)	−0.0033 (13)
C35	0.0377 (18)	0.0281 (17)	0.0377 (19)	0.0022 (15)	−0.0022 (15)	−0.0068 (15)
C36	0.0371 (19)	0.043 (2)	0.0364 (19)	0.0025 (17)	−0.0052 (15)	−0.0114 (16)
C37	0.042 (2)	0.053 (2)	0.0261 (17)	0.0016 (18)	−0.0019 (16)	−0.0059 (16)
C38	0.0412 (19)	0.039 (2)	0.0270 (17)	0.0004 (17)	−0.0025 (15)	0.0027 (15)
C38A	0.0277 (16)	0.0291 (17)	0.0268 (16)	−0.0011 (13)	−0.0015 (13)	0.0023 (13)
C337	0.0336 (16)	0.0178 (14)	0.0268 (16)	0.0008 (13)	0.0005 (13)	0.0026 (12)
O331	0.0517 (14)	0.0184 (11)	0.0251 (11)	−0.0017 (11)	0.0010 (11)	0.0016 (9)
C331	0.0296 (15)	0.0244 (16)	0.0254 (15)	−0.0011 (13)	−0.0001 (13)	0.0033 (12)
C332	0.0324 (17)	0.0255 (16)	0.0319 (17)	0.0009 (14)	−0.0019 (14)	0.0021 (13)
C333	0.045 (2)	0.0304 (18)	0.0345 (18)	0.0046 (16)	−0.0043 (16)	0.0113 (14)
C334	0.055 (2)	0.045 (2)	0.0244 (16)	0.007 (2)	−0.0042 (16)	0.0058 (16)
C335	0.045 (2)	0.038 (2)	0.0295 (17)	0.0029 (17)	−0.0036 (16)	−0.0040 (15)
C336	0.0418 (19)	0.0226 (16)	0.0304 (17)	−0.0010 (15)	0.0016 (15)	0.0016 (13)
C338	0.0391 (19)	0.0221 (16)	0.0406 (18)	0.0006 (15)	−0.0016 (15)	0.0046 (14)
O338	0.087 (2)	0.0234 (14)	0.0528 (17)	0.0118 (14)	−0.0069 (16)	0.0045 (12)
O339	0.0746 (19)	0.0206 (12)	0.0338 (13)	0.0021 (12)	−0.0089 (13)	−0.0035 (10)
C339	0.100 (4)	0.028 (2)	0.047 (2)	0.004 (2)	−0.007 (2)	−0.0121 (18)
C361	0.057 (3)	0.054 (3)	0.045 (2)	0.003 (2)	−0.003 (2)	−0.023 (2)
N41	0.0366 (16)	0.0262 (14)	0.0308 (14)	−0.0005 (12)	−0.0009 (12)	−0.0026 (12)
C42	0.0324 (17)	0.0205 (15)	0.0298 (16)	−0.0022 (13)	−0.0016 (13)	0.0015 (13)
Cl42	0.0676 (6)	0.0203 (4)	0.0306 (4)	−0.0046 (4)	−0.0026 (4)	0.0015 (3)
C43	0.0280 (16)	0.0233 (16)	0.0281 (16)	−0.0012 (13)	−0.0007 (13)	0.0007 (13)
C44	0.0307 (16)	0.0229 (16)	0.0313 (17)	0.0003 (13)	−0.0012 (13)	−0.0051 (13)
C44A	0.0241 (16)	0.0280 (17)	0.0314 (17)	0.0011 (13)	−0.0001 (13)	0.0028 (14)
C45	0.0330 (18)	0.0291 (18)	0.039 (2)	0.0034 (15)	−0.0007 (15)	0.0065 (15)
C46	0.0259 (17)	0.047 (2)	0.039 (2)	0.0050 (16)	−0.0011 (15)	0.0125 (17)
C47	0.040 (2)	0.052 (2)	0.0260 (17)	0.0009 (17)	−0.0003 (15)	0.0090 (16)
C48	0.041 (2)	0.039 (2)	0.0312 (18)	0.0008 (16)	−0.0028 (15)	−0.0050 (16)
C48A	0.0274 (16)	0.0318 (17)	0.0260 (16)	−0.0013 (13)	0.0010 (12)	0.0002 (14)
C437	0.0382 (18)	0.0207 (15)	0.0270 (16)	0.0016 (14)	0.0001 (13)	−0.0025 (13)
O431	0.0513 (15)	0.0210 (12)	0.0278 (12)	0.0010 (11)	−0.0005 (11)	−0.0040 (9)
C431	0.0322 (17)	0.0278 (17)	0.0282 (16)	0.0004 (14)	0.0012 (13)	−0.0039 (13)
C432	0.0329 (18)	0.0305 (18)	0.0307 (17)	−0.0020 (14)	0.0026 (13)	−0.0029 (14)
C433	0.049 (2)	0.0322 (18)	0.0356 (19)	−0.0055 (16)	0.0025 (16)	−0.0107 (16)
C434	0.061 (3)	0.048 (2)	0.0262 (18)	−0.004 (2)	0.0002 (17)	−0.0078 (16)
C435	0.056 (2)	0.041 (2)	0.0294 (19)	0.0004 (19)	0.0032 (17)	0.0040 (16)
C436	0.043 (2)	0.0286 (17)	0.0338 (19)	−0.0036 (16)	0.0031 (15)	−0.0020 (15)
C438	0.0402 (19)	0.0263 (18)	0.0376 (19)	0.0000 (15)	−0.0007 (14)	−0.0065 (14)
O438	0.092 (2)	0.0269 (14)	0.0488 (17)	−0.0053 (15)	−0.0008 (15)	−0.0079 (13)
O439	0.0629 (17)	0.0251 (12)	0.0332 (13)	−0.0066 (11)	−0.0010 (12)	0.0014 (10)
C439	0.078 (3)	0.0304 (19)	0.042 (2)	−0.007 (2)	−0.002 (2)	0.0069 (17)
C461	0.046 (2)	0.059 (3)	0.045 (2)	0.007 (2)	−0.0015 (19)	0.026 (2)

### (III) Methyl 2-[(2-chloro-6-methylquinolin-3-yl)methoxy]benzoate Geometric parameters (Å, º)

**Table:**

N11—C12	1.292 (4)	N31—C32	1.287 (4)
N11—C18A	1.373 (4)	N31—C38A	1.365 (4)
C12—C13	1.412 (4)	C32—C33	1.420 (4)
C12—Cl12	1.752 (3)	C32—Cl32	1.756 (3)
C13—C14	1.375 (4)	C33—C34	1.369 (4)
C13—C137	1.507 (4)	C33—C337	1.507 (4)
C14—C14A	1.408 (4)	C34—C34A	1.409 (4)
C14—H14	0.9500	C34—H34	0.9500
C14A—C18A	1.414 (5)	C34A—C38A	1.415 (5)
C14A—C15	1.421 (5)	C34A—C35	1.418 (5)
C15—C16	1.369 (5)	C35—C36	1.379 (5)
C15—H15	0.9500	C35—H35	0.9500
C16—C17	1.421 (6)	C36—C37	1.415 (6)
C16—C161	1.505 (5)	C36—C361	1.506 (5)
C17—C18	1.370 (5)	C37—C38	1.360 (5)
C17—H17	0.9500	C37—H37	0.9500
C18—C18A	1.411 (5)	C38—C38A	1.422 (5)
C18—H18	0.9500	C38—H38	0.9500
C137—O131	1.419 (4)	C337—O331	1.417 (4)
C137—H13A	0.9900	C337—H33A	0.9900
C137—H13B	0.9900	C337—H33B	0.9900
O131—C131	1.365 (4)	O331—C331	1.355 (4)
C131—C136	1.391 (5)	C331—C336	1.393 (5)
C131—C132	1.408 (5)	C331—C332	1.413 (4)
C132—C133	1.395 (4)	C332—C333	1.392 (5)
C132—C138	1.486 (5)	C332—C338	1.489 (5)
C133—C134	1.378 (5)	C333—C334	1.380 (5)
C133—H133	0.9500	C333—H333	0.9500
C134—C135	1.386 (6)	C334—C335	1.380 (5)
C134—H134	0.9500	C334—H334	0.9500
C135—C136	1.379 (5)	C335—C336	1.383 (5)
C135—H135	0.9500	C335—H335	0.9500
C136—H136	0.9500	C336—H336	0.9500
C138—O138	1.205 (4)	C338—O338	1.205 (5)
C138—O139	1.331 (4)	C338—O339	1.325 (4)
O139—C139	1.437 (4)	O339—C339	1.448 (4)
C139—H19A	0.9800	C339—H39A	0.9800
C139—H19B	0.9800	C339—H39B	0.9800
C139—H19C	0.9800	C339—H39C	0.9800
C161—H16A	0.9800	C361—H36A	0.9800
C161—H16B	0.9800	C361—H36B	0.9800
C161—H16C	0.9800	C361—H36C	0.9800
N21—C22	1.284 (4)	N41—C42	1.289 (4)
N21—C28A	1.367 (5)	N41—C48A	1.366 (4)
C22—C23	1.417 (5)	C42—C43	1.419 (5)
C22—Cl22	1.764 (4)	C42—Cl42	1.757 (3)
C23—C24	1.369 (5)	C43—C44	1.362 (5)
C23—C237	1.498 (5)	C43—C437	1.501 (4)
C24—C24A	1.420 (5)	C44—C44A	1.410 (5)
C24—H24	0.9500	C44—H44	0.9500
C24A—C25	1.415 (5)	C44A—C45	1.417 (5)
C24A—C28A	1.418 (5)	C44A—C48A	1.429 (5)
C25—C26	1.366 (5)	C45—C46	1.368 (5)
C25—H25	0.9500	C45—H45	0.9500
C26—C27	1.413 (6)	C46—C47	1.413 (6)
C26—C261	1.509 (5)	C46—C461	1.519 (5)
C27—C28	1.357 (6)	C47—C48	1.365 (5)
C27—H27	0.9500	C47—H47	0.9500
C28—C28A	1.419 (5)	C48—C48A	1.413 (5)
C28—H28	0.9500	C48—H48	0.9500
C237—O231	1.416 (4)	C437—O431	1.421 (4)
C237—H23A	0.9900	C437—H43A	0.9900
C237—H23B	0.9900	C437—H43B	0.9900
O231—C231	1.359 (4)	O431—C431	1.356 (4)
C231—C236	1.395 (5)	C431—C436	1.400 (5)
C231—C232	1.409 (5)	C431—C432	1.403 (5)
C232—C233	1.394 (5)	C432—C433	1.404 (5)
C232—C238	1.489 (5)	C432—C438	1.494 (5)
C233—C234	1.368 (5)	C433—C434	1.375 (6)
C233—H233	0.9500	C433—H433	0.9500
C234—C235	1.386 (6)	C434—C435	1.384 (6)
C234—H234	0.9500	C434—H434	0.9500
C235—C236	1.384 (5)	C435—C436	1.380 (5)
C235—H235	0.9500	C435—H435	0.9500
C236—H236	0.9500	C436—H436	0.9500
C238—O238	1.195 (4)	C438—O438	1.196 (5)
C238—O239	1.331 (4)	C438—O439	1.340 (4)
O239—C239	1.443 (4)	O439—C439	1.438 (4)
C239—H29A	0.9800	C439—H49A	0.9800
C239—H29B	0.9800	C439—H49B	0.9800
C239—H29C	0.9800	C439—H49C	0.9800
C261—H26A	0.9800	C461—H46A	0.9800
C261—H26B	0.9800	C461—H46B	0.9800
C261—H26C	0.9800	C461—H46C	0.9800
			
C12—N11—C18A	116.9 (3)	C32—N31—C38A	116.9 (3)
N11—C12—C13	127.0 (3)	N31—C32—C33	127.2 (3)
N11—C12—Cl12	115.4 (3)	N31—C32—Cl32	115.7 (2)
C13—C12—Cl12	117.6 (2)	C33—C32—Cl32	117.1 (2)
C14—C13—C12	116.1 (3)	C34—C33—C32	115.8 (3)
C14—C13—C137	123.2 (3)	C34—C33—C337	123.1 (3)
C12—C13—C137	120.7 (3)	C32—C33—C337	121.1 (3)
C13—C14—C14A	120.0 (3)	C33—C34—C34A	120.0 (3)
C13—C14—H14	120.0	C33—C34—H34	120.0
C14A—C14—H14	120.0	C34A—C34—H34	120.0
C14—C14A—C18A	118.2 (3)	C34—C34A—C38A	118.3 (3)
C14—C14A—C15	123.1 (3)	C34—C34A—C35	123.0 (3)
C18A—C14A—C15	118.7 (3)	C38A—C34A—C35	118.8 (3)
C16—C15—C14A	121.4 (3)	C36—C35—C34A	121.5 (3)
C16—C15—H15	119.3	C36—C35—H35	119.3
C14A—C15—H15	119.3	C34A—C35—H35	119.3
C15—C16—C17	118.8 (3)	C35—C36—C37	118.4 (3)
C15—C16—C161	121.8 (4)	C35—C36—C361	120.9 (4)
C17—C16—C161	119.4 (3)	C37—C36—C361	120.6 (3)
C18—C17—C16	121.4 (3)	C38—C37—C36	122.2 (3)
C18—C17—H17	119.3	C38—C37—H37	118.9
C16—C17—H17	119.3	C36—C37—H37	118.9
C17—C18—C18A	119.9 (3)	C37—C38—C38A	119.7 (4)
C17—C18—H18	120.0	C37—C38—H38	120.1
C18A—C18—H18	120.0	C38A—C38—H38	120.1
N11—C18A—C18	118.5 (3)	N31—C38A—C34A	121.8 (3)
N11—C18A—C14A	121.8 (3)	N31—C38A—C38	118.8 (3)
C18—C18A—C14A	119.7 (3)	C34A—C38A—C38	119.4 (3)
O131—C137—C13	107.4 (3)	O331—C337—C33	107.0 (3)
O131—C137—H13A	110.2	O331—C337—H33A	110.3
C13—C137—H13A	110.2	C33—C337—H33A	110.3
O131—C137—H13B	110.2	O331—C337—H33B	110.3
C13—C137—H13B	110.2	C33—C337—H33B	110.3
H13A—C137—H13B	108.5	H33A—C337—H33B	108.6
C131—O131—C137	117.9 (2)	C331—O331—C337	118.5 (2)
O131—C131—C136	122.4 (3)	O331—C331—C336	122.9 (3)
O131—C131—C132	117.6 (3)	O331—C331—C332	117.4 (3)
C136—C131—C132	120.0 (3)	C336—C331—C332	119.7 (3)
C133—C132—C131	117.8 (3)	C333—C332—C331	118.1 (3)
C133—C132—C138	115.4 (3)	C333—C332—C338	115.9 (3)
C131—C132—C138	126.8 (3)	C331—C332—C338	126.1 (3)
C134—C133—C132	122.1 (3)	C334—C333—C332	122.0 (3)
C134—C133—H133	119.0	C334—C333—H333	119.0
C132—C133—H133	119.0	C332—C333—H333	119.0
C133—C134—C135	119.2 (3)	C335—C334—C333	119.2 (3)
C133—C134—H134	120.4	C335—C334—H334	120.4
C135—C134—H134	120.4	C333—C334—H334	120.4
C136—C135—C134	120.4 (3)	C334—C335—C336	120.6 (3)
C136—C135—H135	119.8	C334—C335—H335	119.7
C134—C135—H135	119.8	C336—C335—H335	119.7
C135—C136—C131	120.5 (3)	C335—C336—C331	120.3 (3)
C135—C136—H136	119.8	C335—C336—H336	119.8
C131—C136—H136	119.8	C331—C336—H336	119.8
O138—C138—O139	121.7 (3)	O338—C338—O339	122.7 (3)
O138—C138—C132	122.6 (3)	O338—C338—C332	122.6 (3)
O139—C138—C132	115.6 (3)	O339—C338—C332	114.6 (3)
C138—O139—C139	115.4 (3)	C338—O339—C339	115.2 (3)
O139—C139—H19A	109.5	O339—C339—H39A	109.5
O139—C139—H19B	109.5	O339—C339—H39B	109.5
H19A—C139—H19B	109.5	H39A—C339—H39B	109.5
O139—C139—H19C	109.5	O339—C339—H39C	109.5
H19A—C139—H19C	109.5	H39A—C339—H39C	109.5
H19B—C139—H19C	109.5	H39B—C339—H39C	109.5
C16—C161—H16A	109.5	C36—C361—H36A	109.5
C16—C161—H16B	109.5	C36—C361—H36B	109.5
H16A—C161—H16B	109.5	H36A—C361—H36B	109.5
C16—C161—H16C	109.5	C36—C361—H36C	109.5
H16A—C161—H16C	109.5	H36A—C361—H36C	109.5
H16B—C161—H16C	109.5	H36B—C361—H36C	109.5
C22—N21—C28A	116.7 (3)	C42—N41—C48A	117.0 (3)
N21—C22—C23	127.9 (3)	N41—C42—C43	127.2 (3)
N21—C22—Cl22	115.2 (3)	N41—C42—Cl42	115.6 (3)
C23—C22—Cl22	116.9 (3)	C43—C42—Cl42	117.2 (2)
C24—C23—C22	115.5 (3)	C44—C43—C42	115.9 (3)
C24—C23—C237	123.2 (3)	C44—C43—C437	123.4 (3)
C22—C23—C237	121.3 (3)	C42—C43—C437	120.7 (3)
C23—C24—C24A	120.3 (3)	C43—C44—C44A	120.6 (3)
C23—C24—H24	119.8	C43—C44—H44	119.7
C24A—C24—H24	119.8	C44A—C44—H44	119.7
C25—C24A—C28A	119.0 (3)	C44—C44A—C45	123.8 (3)
C25—C24A—C24	123.4 (3)	C44—C44A—C48A	117.6 (3)
C28A—C24A—C24	117.6 (3)	C45—C44A—C48A	118.5 (3)
C26—C25—C24A	121.3 (4)	C46—C45—C44A	121.5 (4)
C26—C25—H25	119.4	C46—C45—H45	119.3
C24A—C25—H25	119.4	C44A—C45—H45	119.3
C25—C26—C27	118.8 (4)	C45—C46—C47	119.1 (3)
C25—C26—C261	121.1 (4)	C45—C46—C461	121.4 (4)
C27—C26—C261	120.0 (4)	C47—C46—C461	119.5 (4)
C28—C27—C26	122.0 (4)	C48—C47—C46	121.6 (3)
C28—C27—H27	119.0	C48—C47—H47	119.2
C26—C27—H27	119.0	C46—C47—H47	119.2
C27—C28—C28A	119.8 (4)	C47—C48—C48A	120.1 (4)
C27—C28—H28	120.1	C47—C48—H48	119.9
C28A—C28—H28	120.1	C48A—C48—H48	119.9
N21—C28A—C24A	122.0 (3)	N41—C48A—C48	119.2 (3)
N21—C28A—C28	118.9 (3)	N41—C48A—C44A	121.7 (3)
C24A—C28A—C28	119.1 (3)	C48—C48A—C44A	119.1 (3)
O231—C237—C23	107.4 (3)	O431—C437—C43	106.8 (3)
O231—C237—H23A	110.2	O431—C437—H43A	110.4
C23—C237—H23A	110.2	C43—C437—H43A	110.4
O231—C237—H23B	110.2	O431—C437—H43B	110.4
C23—C237—H23B	110.2	C43—C437—H43B	110.4
H23A—C237—H23B	108.5	H43A—C437—H43B	108.6
C231—O231—C237	118.9 (3)	C431—O431—C437	118.5 (3)
O231—C231—C236	122.7 (3)	O431—C431—C436	122.6 (3)
O231—C231—C232	117.5 (3)	O431—C431—C432	118.1 (3)
C236—C231—C232	119.8 (3)	C436—C431—C432	119.4 (3)
C233—C232—C231	117.9 (3)	C431—C432—C433	118.2 (3)
C233—C232—C238	115.7 (3)	C431—C432—C438	126.8 (3)
C231—C232—C238	126.3 (3)	C433—C432—C438	115.0 (3)
C234—C233—C232	122.2 (4)	C434—C433—C432	122.0 (3)
C234—C233—H233	118.9	C434—C433—H433	119.0
C232—C233—H233	118.9	C432—C433—H433	119.0
C233—C234—C235	119.5 (3)	C433—C434—C435	119.2 (3)
C233—C234—H234	120.3	C433—C434—H434	120.4
C235—C234—H234	120.3	C435—C434—H434	120.4
C236—C235—C234	120.2 (3)	C436—C435—C434	120.5 (4)
C236—C235—H235	119.9	C436—C435—H435	119.8
C234—C235—H235	119.9	C434—C435—H435	119.8
C235—C236—C231	120.3 (3)	C435—C436—C431	120.7 (3)
C235—C236—H236	119.8	C435—C436—H436	119.6
C231—C236—H236	119.8	C431—C436—H436	119.6
O238—C238—O239	123.2 (4)	O438—C438—O439	122.7 (4)
O238—C238—C232	122.8 (3)	O438—C438—C432	122.8 (3)
O239—C238—C232	114.0 (3)	O439—C438—C432	114.5 (3)
C238—O239—C239	115.4 (3)	C438—O439—C439	114.6 (3)
O239—C239—H29A	109.5	O439—C439—H49A	109.5
O239—C239—H29B	109.5	O439—C439—H49B	109.5
H29A—C239—H29B	109.5	H49A—C439—H49B	109.5
O239—C239—H29C	109.5	O439—C439—H49C	109.5
H29A—C239—H29C	109.5	H49A—C439—H49C	109.5
H29B—C239—H29C	109.5	H49B—C439—H49C	109.5
C26—C261—H26A	109.5	C46—C461—H46A	109.5
C26—C261—H26B	109.5	C46—C461—H46B	109.5
H26A—C261—H26B	109.5	H46A—C461—H46B	109.5
C26—C261—H26C	109.5	C46—C461—H46C	109.5
H26A—C261—H26C	109.5	H46A—C461—H46C	109.5
H26B—C261—H26C	109.5	H46B—C461—H46C	109.5
			
C18A—N11—C12—C13	1.6 (6)	C38A—N31—C32—C33	−1.5 (5)
C18A—N11—C12—Cl12	−178.1 (2)	C38A—N31—C32—Cl32	177.6 (2)
N11—C12—C13—C14	−1.1 (5)	N31—C32—C33—C34	2.3 (5)
Cl12—C12—C13—C14	178.6 (3)	Cl32—C32—C33—C34	−176.7 (3)
N11—C12—C13—C137	179.3 (3)	N31—C32—C33—C337	−178.2 (3)
Cl12—C12—C13—C137	−1.1 (5)	Cl32—C32—C33—C337	2.8 (4)
C12—C13—C14—C14A	−0.9 (5)	C32—C33—C34—C34A	−1.1 (5)
C137—C13—C14—C14A	178.8 (3)	C337—C33—C34—C34A	179.4 (3)
C13—C14—C14A—C18A	2.1 (5)	C33—C34—C34A—C38A	−0.5 (5)
C13—C14—C14A—C15	−175.6 (3)	C33—C34—C34A—C35	179.8 (3)
C14—C14A—C15—C16	176.9 (4)	C34—C34A—C35—C36	178.9 (3)
C18A—C14A—C15—C16	−0.7 (5)	C38A—C34A—C35—C36	−0.8 (6)
C14A—C15—C16—C17	1.9 (6)	C34A—C35—C36—C37	0.4 (6)
C14A—C15—C16—C161	−178.9 (3)	C34A—C35—C36—C361	−179.9 (4)
C15—C16—C17—C18	−1.5 (6)	C35—C36—C37—C38	−0.1 (6)
C161—C16—C17—C18	179.3 (4)	C361—C36—C37—C38	−179.8 (4)
C16—C17—C18—C18A	−0.1 (6)	C36—C37—C38—C38A	0.2 (6)
C12—N11—C18A—C18	176.9 (3)	C32—N31—C38A—C34A	−0.4 (5)
C12—N11—C18A—C14A	−0.2 (5)	C32—N31—C38A—C38	179.8 (3)
C17—C18—C18A—N11	−175.8 (3)	C34—C34A—C38A—N31	1.4 (5)
C17—C18—C18A—C14A	1.3 (5)	C35—C34A—C38A—N31	−178.9 (3)
C14—C14A—C18A—N11	−1.6 (5)	C34—C34A—C38A—C38	−178.9 (3)
C15—C14A—C18A—N11	176.2 (3)	C35—C34A—C38A—C38	0.9 (5)
C14—C14A—C18A—C18	−178.6 (3)	C37—C38—C38A—N31	179.2 (3)
C15—C14A—C18A—C18	−0.9 (5)	C37—C38—C38A—C34A	−0.6 (6)
C14—C13—C137—O131	0.9 (5)	C34—C33—C337—O331	−2.1 (4)
C12—C13—C137—O131	−179.4 (3)	C32—C33—C337—O331	178.4 (3)
C13—C137—O131—C131	177.2 (3)	C33—C337—O331—C331	−178.9 (3)
C137—O131—C131—C136	3.5 (5)	C337—O331—C331—C336	−1.8 (5)
C137—O131—C131—C132	−176.5 (3)	C337—O331—C331—C332	177.7 (3)
O131—C131—C132—C133	179.1 (3)	O331—C331—C332—C333	178.2 (3)
C136—C131—C132—C133	−0.9 (5)	C336—C331—C332—C333	−2.3 (5)
O131—C131—C132—C138	−1.9 (5)	O331—C331—C332—C338	−1.8 (5)
C136—C131—C132—C138	178.1 (3)	C336—C331—C332—C338	177.7 (3)
C131—C132—C133—C134	0.6 (6)	C331—C332—C333—C334	1.1 (6)
C138—C132—C133—C134	−178.6 (3)	C338—C332—C333—C334	−178.9 (4)
C132—C133—C134—C135	0.3 (6)	C332—C333—C334—C335	0.8 (6)
C133—C134—C135—C136	−0.9 (6)	C333—C334—C335—C336	−1.5 (6)
C134—C135—C136—C131	0.5 (6)	C334—C335—C336—C331	0.2 (6)
O131—C131—C136—C135	−179.6 (3)	O331—C331—C336—C335	−178.9 (3)
C132—C131—C136—C135	0.4 (6)	C332—C331—C336—C335	1.7 (5)
C133—C132—C138—O138	1.6 (6)	C333—C332—C338—O338	11.3 (6)
C131—C132—C138—O138	−177.5 (4)	C331—C332—C338—O338	−168.7 (4)
C133—C132—C138—O139	−178.1 (3)	C333—C332—C338—O339	−167.4 (3)
C131—C132—C138—O139	2.8 (6)	C331—C332—C338—O339	12.7 (5)
O138—C138—O139—C139	−0.5 (6)	O338—C338—O339—C339	−0.3 (6)
C132—C138—O139—C139	179.2 (3)	C332—C338—O339—C339	178.3 (4)
C28A—N21—C22—C23	1.2 (6)	C48A—N41—C42—C43	−0.9 (5)
C28A—N21—C22—Cl22	−177.2 (3)	C48A—N41—C42—Cl42	179.4 (2)
N21—C22—C23—C24	−2.1 (5)	N41—C42—C43—C44	0.0 (5)
Cl22—C22—C23—C24	176.4 (3)	Cl42—C42—C43—C44	179.8 (3)
N21—C22—C23—C237	177.4 (3)	N41—C42—C43—C437	−179.7 (3)
Cl22—C22—C23—C237	−4.2 (4)	Cl42—C42—C43—C437	0.1 (4)
C22—C23—C24—C24A	0.7 (5)	C42—C43—C44—C44A	1.3 (5)
C237—C23—C24—C24A	−178.7 (3)	C437—C43—C44—C44A	−179.0 (3)
C23—C24—C24A—C25	179.7 (3)	C43—C44—C44A—C45	177.5 (3)
C23—C24—C24A—C28A	1.1 (5)	C43—C44—C44A—C48A	−1.7 (5)
C28A—C24A—C25—C26	1.0 (5)	C44—C44A—C45—C46	−180.0 (3)
C24—C24A—C25—C26	−177.5 (4)	C48A—C44A—C45—C46	−0.8 (5)
C24A—C25—C26—C27	−1.1 (6)	C44A—C45—C46—C47	−0.1 (6)
C24A—C25—C26—C261	178.6 (4)	C44A—C45—C46—C461	178.5 (3)
C25—C26—C27—C28	0.1 (6)	C45—C46—C47—C48	0.1 (6)
C261—C26—C27—C28	−179.6 (4)	C461—C46—C47—C48	−178.5 (4)
C26—C27—C28—C28A	0.9 (6)	C46—C47—C48—C48A	0.8 (6)
C22—N21—C28A—C24A	1.0 (5)	C42—N41—C48A—C48	−179.6 (3)
C22—N21—C28A—C28	−179.7 (3)	C42—N41—C48A—C44A	0.4 (5)
C25—C24A—C28A—N21	179.3 (3)	C47—C48—C48A—N41	178.4 (3)
C24—C24A—C28A—N21	−2.1 (5)	C47—C48—C48A—C44A	−1.6 (5)
C25—C24A—C28A—C28	0.0 (5)	C44—C44A—C48A—N41	0.8 (5)
C24—C24A—C28A—C28	178.6 (3)	C45—C44A—C48A—N41	−178.4 (3)
C27—C28—C28A—N21	179.7 (4)	C44—C44A—C48A—C48	−179.2 (3)
C27—C28—C28A—C24A	−1.0 (6)	C45—C44A—C48A—C48	1.6 (5)
C24—C23—C237—O231	−0.8 (5)	C44—C43—C437—O431	2.7 (5)
C22—C23—C237—O231	179.8 (3)	C42—C43—C437—O431	−177.6 (3)
C23—C237—O231—C231	−175.9 (3)	C43—C437—O431—C431	176.4 (3)
C237—O231—C231—C236	−5.8 (5)	C437—O431—C431—C436	4.8 (5)
C237—O231—C231—C232	174.5 (3)	C437—O431—C431—C432	−174.4 (3)
O231—C231—C232—C233	179.3 (3)	O431—C431—C432—C433	−178.1 (3)
C236—C231—C232—C233	−0.3 (5)	C436—C431—C432—C433	2.7 (5)
O231—C231—C232—C238	−1.2 (5)	O431—C431—C432—C438	1.1 (5)
C236—C231—C232—C238	179.2 (3)	C436—C431—C432—C438	−178.1 (3)
C231—C232—C233—C234	0.3 (6)	C431—C432—C433—C434	−1.9 (6)
C238—C232—C233—C234	−179.2 (3)	C438—C432—C433—C434	178.8 (4)
C232—C233—C234—C235	−0.5 (6)	C432—C433—C434—C435	0.6 (6)
C233—C234—C235—C236	0.6 (6)	C433—C434—C435—C436	−0.1 (7)
C234—C235—C236—C231	−0.5 (6)	C434—C435—C436—C431	0.9 (6)
O231—C231—C236—C235	−179.2 (3)	O431—C431—C436—C435	178.5 (4)
C232—C231—C236—C235	0.4 (5)	C432—C431—C436—C435	−2.3 (6)
C233—C232—C238—O238	−14.2 (5)	C431—C432—C438—O438	178.9 (4)
C231—C232—C238—O238	166.4 (4)	C433—C432—C438—O438	−1.9 (6)
C233—C232—C238—O239	164.7 (3)	C431—C432—C438—O439	−0.7 (5)
C231—C232—C238—O239	−14.8 (5)	C433—C432—C438—O439	178.5 (3)
O238—C238—O239—C239	2.5 (6)	O438—C438—O439—C439	0.4 (6)
C232—C238—O239—C239	−176.4 (3)	C432—C438—O439—C439	−180.0 (3)

### (III) Methyl 2-[(2-chloro-6-methylquinolin-3-yl)methoxy]benzoate Hydrogen-bond geometry (Å, º)

**Table:**

*D*—H···*A*	*D*—H	H···*A*	*D*···*A*	*D*—H···*A*
C28—H28···N41^i^	0.95	2.63	3.565 (5)	169
C136—H136···O138^ii^	0.95	2.50	3.261 (4)	137
C236—H236···O438^iii^	0.95	2.43	3.223 (4)	141
C336—H336···O338^iv^	0.95	2.46	3.238 (4)	139
C436—H436···O238^iii^	0.95	2.51	3.254 (4)	136
C337—H33*B*···*Cg*1	0.99	2.64	3.441 (4)	138
C437—H43*A*···*Cg*2	0.99	2.64	3.446 (4)	138

Symmetry codes: (i) *x*−1/2, −*y*+3/2, −*z*+1; (ii) −*x*, *y*−1/2, −*z*+1/2; (iii) −*x*+1, *y*+1/2, −*z*+1/2; (iv) −*x*+1, *y*−1/2, −*z*+1/2.

### (IV) 2-Chloro-3-[(naphthalen-1-yloxy)methyl]quinoline Crystal data

**Table:**

C_20_H_14_ClNO	*F*(000) = 332
*M**_r_* = 319.77	*D*_x_ = 1.411 Mg m^−^^3^
Monoclinic, *P*2_1_	Cu *K*α radiation, λ = 1.54184 Å
*a* = 5.3165 (3) Å	Cell parameters from 2014 reflections
*b* = 10.5098 (4) Å	θ = 3.3–72.6°
*c* = 13.6201 (7) Å	µ = 2.27 mm^−^^1^
β = 98.527 (5)°	*T* = 173 K
*V* = 752.62 (6) Å^3^	Needle, colourless
*Z* = 2	0.34 × 0.10 × 0.08 mm

### (IV) 2-Chloro-3-[(naphthalen-1-yloxy)methyl]quinoline Data collection

**Table:**

Agilent Eos Gemini diffractometer	1938 reflections with *I* > 2σ(*I*)
Radiation source: Enhance (Cu) X-ray Source	*R*_int_ = 0.029
ω scans	θ_max_ = 72.6°, θ_min_ = 3.3°
Absorption correction: multi-scan (*SADABS*; Sheldrick, 2003)	*h* = −6→6
*T*_min_ = 0.551, *T*_max_ = 0.834	*k* = −8→12
4606 measured reflections	*l* = −16→16
2014 independent reflections	

### (IV) 2-Chloro-3-[(naphthalen-1-yloxy)methyl]quinoline Refinement

**Table:**

Refinement on *F*^2^	Hydrogen site location: inferred from neighbouring sites
Least-squares matrix: full	H-atom parameters constrained
*R*[*F*^2^ > 2σ(*F*^2^)] = 0.033	*w* = 1/[σ^2^(*F*_o_^2^) + (0.0579*P*)^2^] where *P* = (*F*_o_^2^ + 2*F*_c_^2^)/3
*wR*(*F*^2^) = 0.090	(Δ/σ)_max_ < 0.001
*S* = 1.08	Δρ_max_ = 0.22 e Å^−^^3^
2014 reflections	Δρ_min_ = −0.19 e Å^−^^3^
208 parameters	Absolute structure: Classical Flack method preferred over Parsons because s.u. lower.
1 restraint	Absolute structure parameter: −0.007 (18)

### (IV) 2-Chloro-3-[(naphthalen-1-yloxy)methyl]quinoline Special details

**Table:**

Geometry. All e.s.d.'s (except the e.s.d. in the dihedral angle between two l.s. planes) are estimated using the full covariance matrix. The cell e.s.d.'s are taken into account individually in the estimation of e.s.d.'s in distances, angles and torsion angles; correlations between e.s.d.'s in cell parameters are only used when they are defined by crystal symmetry. An approximate (isotropic) treatment of cell e.s.d.'s is used for estimating e.s.d.'s involving l.s. planes.

### (IV) 2-Chloro-3-[(naphthalen-1-yloxy)methyl]quinoline Fractional atomic coordinates and isotropic or equivalent isotropic displacement parameters (Å^2^)

**Table:**

	*x*	*y*	*z*	*U*_iso_*/*U*_eq_	
N1	0.1678 (4)	0.8551 (2)	0.36307 (18)	0.0324 (5)	
C2	0.3087 (5)	0.8457 (3)	0.29394 (19)	0.0300 (5)	
Cl2	0.25067 (12)	0.95854 (7)	0.19826 (5)	0.04033 (19)	
C3	0.5025 (5)	0.7539 (2)	0.28753 (19)	0.0291 (5)	
C4	0.5420 (5)	0.6680 (3)	0.3636 (2)	0.0311 (5)	
H4	0.6701	0.6049	0.3641	0.037*	
C4A	0.3943 (5)	0.6721 (3)	0.4417 (2)	0.0301 (5)	
C5	0.4220 (6)	0.5842 (3)	0.5210 (2)	0.0373 (6)	
H5	0.5494	0.5202	0.5244	0.045*	
C6	0.2686 (6)	0.5897 (3)	0.5927 (2)	0.0392 (6)	
H6	0.2863	0.5284	0.6446	0.047*	
C7	0.0835 (5)	0.6863 (3)	0.5899 (2)	0.0394 (6)	
H7	−0.0216	0.6903	0.6404	0.047*	
C8	0.0539 (5)	0.7742 (3)	0.5151 (2)	0.0375 (6)	
H8	−0.0698	0.8396	0.5145	0.045*	
C8A	0.2063 (5)	0.7682 (2)	0.4390 (2)	0.0301 (5)	
C37A	0.6440 (5)	0.7514 (3)	0.20041 (19)	0.0309 (5)	
H37A	0.5238	0.7395	0.1383	0.037*	
H37B	0.7351	0.8329	0.1958	0.037*	
O31	0.8208 (4)	0.64868 (19)	0.21392 (13)	0.0335 (4)	
C31	0.9563 (5)	0.6249 (3)	0.13811 (19)	0.0300 (5)	
C32	0.9293 (5)	0.6908 (3)	0.0506 (2)	0.0338 (6)	
H32	0.8081	0.7576	0.0391	0.041*	
C33	1.0815 (5)	0.6599 (3)	−0.0230 (2)	0.0359 (6)	
H33	1.0579	0.7047	−0.0842	0.043*	
C34	1.2606 (5)	0.5671 (3)	−0.0073 (2)	0.0372 (6)	
H34	1.3650	0.5496	−0.0566	0.045*	
C34A	1.2927 (5)	0.4962 (2)	0.0824 (2)	0.0323 (6)	
C35	1.4795 (5)	0.3997 (3)	0.1028 (2)	0.0375 (6)	
H35	1.5873	0.3812	0.0550	0.045*	
C36	1.5078 (5)	0.3334 (3)	0.1892 (2)	0.0417 (7)	
H36	1.6336	0.2687	0.2007	0.050*	
C37	1.3522 (5)	0.3594 (3)	0.2622 (2)	0.0386 (6)	
H37	1.3739	0.3127	0.3226	0.046*	
C38	1.1692 (5)	0.4525 (3)	0.24564 (19)	0.0329 (5)	
H38	1.0628	0.4690	0.2944	0.040*	
C38A	1.1379 (5)	0.5238 (2)	0.1565 (2)	0.0288 (5)	

### (IV) 2-Chloro-3-[(naphthalen-1-yloxy)methyl]quinoline Atomic displacement parameters (Å^2^)

**Table:**

	*U*^11^	*U*^22^	*U*^33^	*U*^12^	*U*^13^	*U*^23^
N1	0.0306 (11)	0.0295 (11)	0.0363 (11)	0.0035 (9)	0.0022 (9)	−0.0015 (9)
C2	0.0298 (12)	0.0270 (12)	0.0313 (13)	−0.0002 (10)	−0.0016 (10)	0.0006 (10)
Cl2	0.0429 (3)	0.0359 (3)	0.0417 (3)	0.0079 (3)	0.0048 (2)	0.0091 (3)
C3	0.0259 (12)	0.0279 (12)	0.0319 (12)	−0.0018 (10)	−0.0011 (10)	−0.0042 (10)
C4	0.0276 (12)	0.0281 (12)	0.0365 (13)	0.0036 (10)	0.0016 (10)	−0.0036 (11)
C4A	0.0268 (11)	0.0267 (12)	0.0355 (13)	−0.0020 (10)	0.0007 (10)	−0.0045 (10)
C5	0.0376 (13)	0.0330 (14)	0.0403 (14)	0.0040 (11)	0.0029 (11)	0.0025 (11)
C6	0.0407 (15)	0.0389 (16)	0.0369 (14)	−0.0042 (13)	0.0023 (12)	0.0053 (12)
C7	0.0346 (14)	0.0493 (17)	0.0356 (14)	−0.0025 (13)	0.0090 (11)	−0.0027 (12)
C8	0.0321 (14)	0.0393 (15)	0.0410 (15)	0.0034 (12)	0.0050 (11)	−0.0038 (12)
C8A	0.0265 (11)	0.0286 (12)	0.0339 (13)	−0.0017 (10)	0.0006 (10)	−0.0046 (10)
C37A	0.0287 (12)	0.0285 (12)	0.0348 (13)	0.0013 (10)	0.0024 (10)	0.0010 (10)
O31	0.0363 (9)	0.0332 (10)	0.0320 (9)	0.0074 (8)	0.0078 (7)	0.0022 (7)
C31	0.0281 (12)	0.0308 (12)	0.0313 (12)	−0.0043 (10)	0.0053 (10)	−0.0024 (10)
C32	0.0327 (13)	0.0349 (14)	0.0331 (13)	0.0000 (11)	0.0027 (10)	−0.0005 (11)
C33	0.0386 (14)	0.0397 (15)	0.0287 (13)	−0.0083 (12)	0.0031 (11)	0.0006 (11)
C34	0.0365 (14)	0.0416 (15)	0.0348 (14)	−0.0083 (12)	0.0095 (11)	−0.0087 (12)
C34A	0.0290 (12)	0.0316 (14)	0.0355 (13)	−0.0061 (10)	0.0024 (10)	−0.0088 (10)
C35	0.0298 (13)	0.0373 (14)	0.0466 (15)	−0.0011 (11)	0.0095 (11)	−0.0108 (12)
C36	0.0313 (14)	0.0358 (15)	0.0571 (18)	0.0044 (12)	0.0033 (13)	−0.0027 (13)
C37	0.0358 (14)	0.0366 (14)	0.0423 (15)	0.0008 (12)	0.0023 (12)	0.0058 (13)
C38	0.0301 (11)	0.0324 (13)	0.0367 (12)	−0.0015 (12)	0.0063 (10)	−0.0015 (13)
C38A	0.0259 (11)	0.0270 (12)	0.0327 (12)	−0.0043 (9)	0.0013 (10)	−0.0039 (10)

### (IV) 2-Chloro-3-[(naphthalen-1-yloxy)methyl]quinoline Geometric parameters (Å, º)

**Table:**

N1—C2	1.291 (3)	C37A—H37B	0.9900
N1—C8A	1.372 (3)	O31—C31	1.367 (3)
C2—C3	1.424 (4)	C31—C32	1.368 (4)
C2—Cl2	1.755 (3)	C31—C38A	1.432 (4)
C3—C4	1.367 (4)	C32—C33	1.416 (4)
C3—C37A	1.497 (3)	C32—H32	0.9500
C4—C4A	1.413 (3)	C33—C34	1.357 (5)
C4—H4	0.9500	C33—H33	0.9500
C4A—C5	1.413 (4)	C34—C34A	1.420 (4)
C4A—C8A	1.418 (4)	C34—H34	0.9500
C5—C6	1.364 (4)	C34A—C35	1.417 (4)
C5—H5	0.9500	C34A—C38A	1.423 (4)
C6—C7	1.410 (4)	C35—C36	1.357 (4)
C6—H6	0.9500	C35—H35	0.9500
C7—C8	1.366 (4)	C36—C37	1.411 (4)
C7—H7	0.9500	C36—H36	0.9500
C8—C8A	1.409 (4)	C37—C38	1.375 (4)
C8—H8	0.9500	C37—H37	0.9500
C37A—O31	1.426 (3)	C38—C38A	1.416 (4)
C37A—H37A	0.9900	C38—H38	0.9500
			
C2—N1—C8A	117.4 (2)	H37A—C37A—H37B	108.4
N1—C2—C3	126.9 (2)	C31—O31—C37A	116.9 (2)
N1—C2—Cl2	115.61 (19)	O31—C31—C32	124.5 (2)
C3—C2—Cl2	117.5 (2)	O31—C31—C38A	114.9 (2)
C4—C3—C2	115.6 (2)	C32—C31—C38A	120.6 (2)
C4—C3—C37A	123.4 (2)	C31—C32—C33	120.2 (3)
C2—C3—C37A	120.9 (2)	C31—C32—H32	119.9
C3—C4—C4A	120.7 (2)	C33—C32—H32	119.9
C3—C4—H4	119.7	C34—C33—C32	121.0 (3)
C4A—C4—H4	119.7	C34—C33—H33	119.5
C4—C4A—C5	123.3 (2)	C32—C33—H33	119.5
C4—C4A—C8A	118.1 (2)	C33—C34—C34A	120.3 (2)
C5—C4A—C8A	118.6 (2)	C33—C34—H34	119.9
C6—C5—C4A	120.9 (3)	C34A—C34—H34	119.9
C6—C5—H5	119.5	C35—C34A—C34	122.4 (3)
C4A—C5—H5	119.5	C35—C34A—C38A	118.0 (3)
C5—C6—C7	120.0 (3)	C34—C34A—C38A	119.6 (3)
C5—C6—H6	120.0	C36—C35—C34A	121.5 (3)
C7—C6—H6	120.0	C36—C35—H35	119.3
C8—C7—C6	120.7 (3)	C34A—C35—H35	119.3
C8—C7—H7	119.7	C35—C36—C37	120.7 (3)
C6—C7—H7	119.7	C35—C36—H36	119.7
C7—C8—C8A	120.2 (3)	C37—C36—H36	119.7
C7—C8—H8	119.9	C38—C37—C36	119.8 (3)
C8A—C8—H8	119.9	C38—C37—H37	120.1
N1—C8A—C8	119.2 (2)	C36—C37—H37	120.1
N1—C8A—C4A	121.3 (2)	C37—C38—C38A	120.5 (2)
C8—C8A—C4A	119.5 (2)	C37—C38—H38	119.7
O31—C37A—C3	108.1 (2)	C38A—C38—H38	119.7
O31—C37A—H37A	110.1	C38—C38A—C34A	119.5 (2)
C3—C37A—H37A	110.1	C38—C38A—C31	122.2 (2)
O31—C37A—H37B	110.1	C34A—C38A—C31	118.2 (2)
C3—C37A—H37B	110.1		
			
C8A—N1—C2—C3	0.0 (4)	C3—C37A—O31—C31	175.4 (2)
C8A—N1—C2—Cl2	179.65 (19)	C37A—O31—C31—C32	−1.4 (4)
N1—C2—C3—C4	−0.5 (4)	C37A—O31—C31—C38A	177.9 (2)
Cl2—C2—C3—C4	179.83 (19)	O31—C31—C32—C33	179.2 (3)
N1—C2—C3—C37A	177.5 (2)	C38A—C31—C32—C33	−0.1 (4)
Cl2—C2—C3—C37A	−2.1 (3)	C31—C32—C33—C34	−1.7 (4)
C2—C3—C4—C4A	0.7 (4)	C32—C33—C34—C34A	2.1 (4)
C37A—C3—C4—C4A	−177.3 (2)	C33—C34—C34A—C35	−179.0 (2)
C3—C4—C4A—C5	178.4 (3)	C33—C34—C34A—C38A	−0.7 (4)
C3—C4—C4A—C8A	−0.5 (4)	C34—C34A—C35—C36	179.7 (3)
C4—C4A—C5—C6	−177.7 (3)	C38A—C34A—C35—C36	1.4 (4)
C8A—C4A—C5—C6	1.2 (4)	C34A—C35—C36—C37	−0.6 (4)
C4A—C5—C6—C7	−1.7 (4)	C35—C36—C37—C38	0.3 (4)
C5—C6—C7—C8	0.7 (5)	C36—C37—C38—C38A	−0.9 (4)
C6—C7—C8—C8A	0.9 (4)	C37—C38—C38A—C34A	1.8 (4)
C2—N1—C8A—C8	−179.1 (2)	C37—C38—C38A—C31	−177.5 (2)
C2—N1—C8A—C4A	0.3 (4)	C35—C34A—C38A—C38	−2.0 (4)
C7—C8—C8A—N1	178.0 (3)	C34—C34A—C38A—C38	179.6 (2)
C7—C8—C8A—C4A	−1.4 (4)	C35—C34A—C38A—C31	177.3 (2)
C4—C4A—C8A—N1	−0.1 (4)	C34—C34A—C38A—C31	−1.0 (3)
C5—C4A—C8A—N1	−179.0 (2)	O31—C31—C38A—C38	1.4 (3)
C4—C4A—C8A—C8	179.3 (2)	C32—C31—C38A—C38	−179.2 (2)
C5—C4A—C8A—C8	0.4 (4)	O31—C31—C38A—C34A	−177.9 (2)
C4—C3—C37A—O31	−1.1 (3)	C32—C31—C38A—C34A	1.4 (3)
C2—C3—C37A—O31	−179.0 (2)		

### (IV) 2-Chloro-3-[(naphthalen-1-yloxy)methyl]quinoline Hydrogen-bond geometry (Å, º)

**Table:**

*D*—H···*A*	*D*—H	H···*A*	*D*···*A*	*D*—H···*A*
C37*A*—H37*A*···*Cg*3^i^	0.99	2.74	3.552 (3)	139

Symmetry code: (i) *x*−1, *y*, *z*.

### (V) {5-[(2-Chloroquinolin-3-yl)methoxy]-4-(hydroxymethyl)-6-methyl-pyridin-3-yl}methanol Crystal data

**Table:**

C_18_H_17_ClN_2_O_3_	*F*(000) = 720
*M**_r_* = 344.79	*D*_x_ = 1.433 Mg m^−^^3^
Monoclinic, *P*2_1_/*n*	Cu *K*α radiation, λ = 1.54184 Å
*a* = 9.7866 (3) Å	Cell parameters from 3112 reflections
*b* = 15.3336 (4) Å	θ = 5.1–72.5°
*c* = 10.6570 (3) Å	µ = 2.29 mm^−^^1^
β = 92.381 (3)°	*T* = 173 K
*V* = 1597.85 (8) Å^3^	Block, colourless
*Z* = 4	0.42 × 0.38 × 0.32 mm

### (V) {5-[(2-Chloroquinolin-3-yl)methoxy]-4-(hydroxymethyl)-6-methyl-pyridin-3-yl}methanol Data collection

**Table:**

Agilent Eos Gemini diffractometer	2764 reflections with *I* > 2σ(*I*)
Radiation source: Enhance (Cu) X-ray Source	*R*_int_ = 0.043
ω scans	θ_max_ = 72.5°, θ_min_ = 5.1°
Absorption correction: multi-scan (*SADABS*; Sheldrick, 2003)	*h* = −11→9
*T*_min_ = 0.375, *T*_max_ = 0.481	*k* = −17→18
9423 measured reflections	*l* = −13→12
3112 independent reflections	

### (V) {5-[(2-Chloroquinolin-3-yl)methoxy]-4-(hydroxymethyl)-6-methyl-pyridin-3-yl}methanol Refinement

**Table:**

Refinement on *F*^2^	Hydrogen site location: mixed
Least-squares matrix: full	H-atom parameters constrained
*R*[*F*^2^ > 2σ(*F*^2^)] = 0.045	*w* = 1/[σ^2^(*F*_o_^2^) + (0.0793*P*)^2^ + 0.2783*P*] where *P* = (*F*_o_^2^ + 2*F*_c_^2^)/3
*wR*(*F*^2^) = 0.127	(Δ/σ)_max_ = 0.001
*S* = 1.05	Δρ_max_ = 0.32 e Å^−^^3^
3112 reflections	Δρ_min_ = −0.25 e Å^−^^3^
219 parameters	Extinction correction: *SHELXL2014* (Sheldrick, 2015), Fc^*^=kFc[1+0.001xFc^2^λ^3^/sin(2θ)]^-1/4^
0 restraints	Extinction coefficient: 0.0022 (4)

### (V) {5-[(2-Chloroquinolin-3-yl)methoxy]-4-(hydroxymethyl)-6-methyl-pyridin-3-yl}methanol Special details

**Table:**

Geometry. All e.s.d.'s (except the e.s.d. in the dihedral angle between two l.s. planes) are estimated using the full covariance matrix. The cell e.s.d.'s are taken into account individually in the estimation of e.s.d.'s in distances, angles and torsion angles; correlations between e.s.d.'s in cell parameters are only used when they are defined by crystal symmetry. An approximate (isotropic) treatment of cell e.s.d.'s is used for estimating e.s.d.'s involving l.s. planes.

### (V) {5-[(2-Chloroquinolin-3-yl)methoxy]-4-(hydroxymethyl)-6-methyl-pyridin-3-yl}methanol Fractional atomic coordinates and isotropic or equivalent isotropic displacement parameters (Å^2^)

**Table:**

	*x*	*y*	*z*	*U*_iso_*/*U*_eq_	
N1	0.14131 (14)	0.33261 (9)	0.62337 (13)	0.0316 (3)	
C2	0.16152 (16)	0.41138 (10)	0.58407 (15)	0.0285 (3)	
Cl2	0.10286 (4)	0.49427 (3)	0.68208 (4)	0.03973 (18)	
C3	0.22557 (16)	0.43634 (10)	0.47234 (16)	0.0289 (3)	
C4	0.27768 (17)	0.36931 (11)	0.40445 (15)	0.0317 (4)	
H4	0.3241	0.3818	0.3300	0.038*	
C4A	0.26375 (17)	0.28188 (11)	0.44305 (16)	0.0310 (4)	
C5	0.3168 (2)	0.21043 (12)	0.37582 (18)	0.0389 (4)	
H5	0.3677	0.2203	0.3031	0.047*	
C6	0.2941 (2)	0.12723 (12)	0.41650 (19)	0.0429 (5)	
H6	0.3305	0.0795	0.3720	0.052*	
C7	0.2183 (2)	0.11136 (12)	0.52249 (19)	0.0463 (5)	
H7	0.2022	0.0530	0.5479	0.056*	
C8	0.1668 (2)	0.17907 (12)	0.59013 (17)	0.0408 (4)	
H8	0.1155	0.1677	0.6621	0.049*	
C8A	0.19062 (18)	0.26579 (10)	0.55190 (16)	0.0313 (4)	
C37	0.23683 (17)	0.52862 (11)	0.42748 (17)	0.0329 (4)	
H37A	0.2336	0.5302	0.3345	0.039*	
H37B	0.1596	0.5636	0.4573	0.039*	
O31	0.36442 (11)	0.56428 (7)	0.47603 (10)	0.0276 (3)	
N31	0.51999 (13)	0.70720 (9)	0.25268 (13)	0.0297 (3)	
C32	0.47930 (15)	0.63485 (10)	0.30994 (15)	0.0259 (3)	
C33	0.39991 (15)	0.64051 (10)	0.41634 (14)	0.0236 (3)	
C34	0.36223 (15)	0.72095 (10)	0.46347 (14)	0.0258 (3)	
C35	0.40670 (16)	0.79604 (10)	0.40161 (15)	0.0284 (3)	
C36	0.48433 (17)	0.78469 (10)	0.29806 (16)	0.0311 (4)	
H36	0.5145	0.8354	0.2561	0.037*	
C321	0.52007 (17)	0.54887 (11)	0.25592 (17)	0.0329 (4)	
H32A	0.5987	0.5572	0.2032	0.039*	
H32B	0.4434	0.5249	0.2049	0.039*	
H32C	0.5447	0.5084	0.3243	0.039*	
C341	0.27452 (17)	0.72795 (11)	0.57607 (15)	0.0316 (4)	
H41A	0.2588	0.6694	0.6119	0.038*	
H41B	0.3204	0.7647	0.6416	0.038*	
O341	0.14772 (13)	0.76632 (10)	0.53514 (12)	0.0437 (3)	
H341	0.1027	0.7791	0.6056	0.066*	
C351	0.36913 (19)	0.88673 (11)	0.44319 (18)	0.0371 (4)	
H51A	0.3812	0.8906	0.5357	0.045*	
H51B	0.4321	0.9293	0.4063	0.045*	
O351	0.23190 (14)	0.90946 (8)	0.40706 (13)	0.0434 (3)	
H351	0.1838	0.8642	0.4382	0.065*	

### (V) {5-[(2-Chloroquinolin-3-yl)methoxy]-4-(hydroxymethyl)-6-methyl-pyridin-3-yl}methanol Atomic displacement parameters (Å^2^)

**Table:**

	*U*^11^	*U*^22^	*U*^33^	*U*^12^	*U*^13^	*U*^23^
N1	0.0401 (7)	0.0246 (7)	0.0303 (7)	−0.0041 (5)	0.0016 (6)	0.0012 (5)
C2	0.0320 (7)	0.0220 (7)	0.0314 (8)	−0.0007 (6)	0.0000 (6)	−0.0026 (6)
Cl2	0.0436 (3)	0.0287 (2)	0.0476 (3)	0.00182 (15)	0.0100 (2)	−0.00770 (16)
C3	0.0312 (8)	0.0235 (8)	0.0318 (8)	−0.0028 (6)	−0.0022 (6)	0.0030 (6)
C4	0.0380 (8)	0.0295 (9)	0.0274 (8)	−0.0038 (6)	0.0000 (6)	0.0017 (6)
C4A	0.0377 (8)	0.0258 (8)	0.0290 (8)	−0.0007 (6)	−0.0044 (7)	−0.0014 (6)
C5	0.0460 (10)	0.0349 (9)	0.0353 (9)	0.0023 (7)	−0.0043 (8)	−0.0076 (7)
C6	0.0575 (11)	0.0270 (9)	0.0430 (10)	0.0059 (8)	−0.0132 (9)	−0.0105 (7)
C7	0.0708 (13)	0.0214 (8)	0.0453 (11)	−0.0039 (8)	−0.0149 (10)	−0.0006 (7)
C8	0.0602 (11)	0.0266 (9)	0.0350 (9)	−0.0081 (8)	−0.0051 (8)	0.0024 (7)
C8A	0.0413 (9)	0.0233 (8)	0.0285 (8)	−0.0029 (6)	−0.0068 (7)	0.0009 (6)
C37	0.0346 (8)	0.0255 (8)	0.0381 (9)	−0.0030 (6)	−0.0039 (7)	0.0081 (7)
O31	0.0319 (6)	0.0216 (5)	0.0292 (6)	−0.0012 (4)	0.0000 (4)	0.0060 (4)
N31	0.0299 (7)	0.0274 (7)	0.0324 (7)	−0.0012 (5)	0.0082 (5)	0.0014 (5)
C32	0.0255 (7)	0.0240 (8)	0.0280 (8)	0.0017 (6)	0.0018 (6)	−0.0011 (6)
C33	0.0254 (7)	0.0204 (7)	0.0251 (7)	−0.0004 (5)	0.0005 (6)	0.0025 (5)
C34	0.0284 (7)	0.0250 (8)	0.0240 (7)	0.0007 (5)	0.0012 (6)	−0.0002 (6)
C35	0.0333 (8)	0.0201 (7)	0.0318 (8)	−0.0005 (6)	0.0025 (6)	−0.0011 (6)
C36	0.0331 (8)	0.0256 (8)	0.0351 (9)	−0.0033 (6)	0.0064 (6)	0.0038 (6)
C321	0.0363 (8)	0.0274 (8)	0.0354 (9)	0.0056 (6)	0.0058 (7)	−0.0048 (7)
C341	0.0407 (9)	0.0304 (8)	0.0244 (8)	0.0032 (6)	0.0084 (6)	−0.0011 (6)
O341	0.0404 (7)	0.0575 (8)	0.0343 (7)	0.0124 (6)	0.0145 (5)	0.0018 (6)
C351	0.0481 (10)	0.0220 (8)	0.0415 (10)	0.0018 (7)	0.0067 (8)	−0.0035 (7)
O351	0.0531 (8)	0.0307 (7)	0.0467 (8)	0.0135 (6)	0.0046 (6)	−0.0013 (5)

### (V) {5-[(2-Chloroquinolin-3-yl)methoxy]-4-(hydroxymethyl)-6-methyl-pyridin-3-yl}methanol Geometric parameters (Å, º)

**Table:**

N1—C2	1.296 (2)	N31—C36	1.335 (2)
N1—C8A	1.376 (2)	N31—C32	1.335 (2)
C2—C3	1.421 (2)	C32—C33	1.404 (2)
C2—Cl2	1.7566 (16)	C32—C321	1.499 (2)
C3—C4	1.368 (2)	C33—C34	1.388 (2)
C3—C37	1.499 (2)	C34—C35	1.405 (2)
C4—C4A	1.411 (2)	C34—C341	1.508 (2)
C4—H4	0.9500	C35—C36	1.377 (2)
C4A—C8A	1.410 (2)	C35—C351	1.510 (2)
C4A—C5	1.419 (2)	C36—H36	0.9500
C5—C6	1.368 (3)	C321—H32A	0.9800
C5—H5	0.9500	C321—H32B	0.9800
C6—C7	1.398 (3)	C321—H32C	0.9800
C6—H6	0.9500	C341—O341	1.425 (2)
C7—C8	1.372 (3)	C341—H41A	0.9900
C7—H7	0.9500	C341—H41B	0.9900
C8—C8A	1.413 (2)	O341—H341	0.9077
C8—H8	0.9500	C351—O351	1.425 (2)
C37—O31	1.4394 (19)	C351—H51A	0.9900
C37—H37A	0.9900	C351—H51B	0.9900
C37—H37B	0.9900	O351—H351	0.9093
O31—C33	1.3819 (18)		
			
C2—N1—C8A	116.95 (14)	N31—C32—C33	120.23 (14)
N1—C2—C3	126.88 (15)	N31—C32—C321	117.78 (14)
N1—C2—Cl2	115.09 (12)	C33—C32—C321	121.98 (14)
C3—C2—Cl2	118.02 (12)	O31—C33—C34	120.64 (14)
C4—C3—C2	115.34 (14)	O31—C33—C32	118.54 (13)
C4—C3—C37	120.45 (15)	C34—C33—C32	120.78 (13)
C2—C3—C37	124.21 (15)	C33—C34—C35	117.81 (14)
C3—C4—C4A	121.15 (15)	C33—C34—C341	121.33 (14)
C3—C4—H4	119.4	C35—C34—C341	120.85 (14)
C4A—C4—H4	119.4	C36—C35—C34	117.68 (14)
C8A—C4A—C4	117.69 (15)	C36—C35—C351	120.08 (15)
C8A—C4A—C5	119.29 (15)	C34—C35—C351	122.22 (15)
C4—C4A—C5	123.00 (16)	N31—C36—C35	124.36 (14)
C6—C5—C4A	119.48 (18)	N31—C36—H36	117.8
C6—C5—H5	120.3	C35—C36—H36	117.8
C4A—C5—H5	120.3	C32—C321—H32A	109.5
C5—C6—C7	121.11 (17)	C32—C321—H32B	109.5
C5—C6—H6	119.4	H32A—C321—H32B	109.5
C7—C6—H6	119.4	C32—C321—H32C	109.5
C8—C7—C6	120.77 (17)	H32A—C321—H32C	109.5
C8—C7—H7	119.6	H32B—C321—H32C	109.5
C6—C7—H7	119.6	O341—C341—C34	107.64 (13)
C7—C8—C8A	119.49 (18)	O341—C341—H41A	110.2
C7—C8—H8	120.3	C34—C341—H41A	110.2
C8A—C8—H8	120.3	O341—C341—H41B	110.2
N1—C8A—C4A	121.79 (15)	C34—C341—H41B	110.2
N1—C8A—C8	118.38 (16)	H41A—C341—H41B	108.5
C4A—C8A—C8	119.83 (16)	C341—O341—H341	106.4
O31—C37—C3	108.55 (12)	O351—C351—C35	112.61 (14)
O31—C37—H37A	110.0	O351—C351—H51A	109.1
C3—C37—H37A	110.0	C35—C351—H51A	109.1
O31—C37—H37B	110.0	O351—C351—H51B	109.1
C3—C37—H37B	110.0	C35—C351—H51B	109.1
H37A—C37—H37B	108.4	H51A—C351—H51B	107.8
C33—O31—C37	112.75 (11)	C351—O351—H351	102.2
C36—N31—C32	119.13 (14)		
			
C8A—N1—C2—C3	2.2 (2)	C3—C37—O31—C33	165.21 (13)
C8A—N1—C2—Cl2	−177.48 (11)	C36—N31—C32—C33	0.1 (2)
N1—C2—C3—C4	−4.1 (2)	C36—N31—C32—C321	179.48 (14)
Cl2—C2—C3—C4	175.57 (12)	C37—O31—C33—C34	92.25 (17)
N1—C2—C3—C37	175.40 (15)	C37—O31—C33—C32	−90.17 (17)
Cl2—C2—C3—C37	−4.9 (2)	N31—C32—C33—O31	−177.42 (13)
C2—C3—C4—C4A	1.8 (2)	C321—C32—C33—O31	3.2 (2)
C37—C3—C4—C4A	−177.78 (14)	N31—C32—C33—C34	0.2 (2)
C3—C4—C4A—C8A	1.9 (2)	C321—C32—C33—C34	−179.21 (14)
C3—C4—C4A—C5	−179.68 (15)	O31—C33—C34—C35	177.22 (13)
C8A—C4A—C5—C6	1.1 (3)	C32—C33—C34—C35	−0.3 (2)
C4—C4A—C5—C6	−177.29 (16)	O31—C33—C34—C341	−3.8 (2)
C4A—C5—C6—C7	0.7 (3)	C32—C33—C34—C341	178.71 (14)
C5—C6—C7—C8	−1.3 (3)	C33—C34—C35—C36	0.2 (2)
C6—C7—C8—C8A	0.1 (3)	C341—C34—C35—C36	−178.80 (14)
C2—N1—C8A—C4A	2.0 (2)	C33—C34—C35—C351	178.66 (14)
C2—N1—C8A—C8	−178.19 (15)	C341—C34—C35—C351	−0.4 (2)
C4—C4A—C8A—N1	−4.0 (2)	C32—N31—C36—C35	−0.2 (3)
C5—C4A—C8A—N1	177.53 (15)	C34—C35—C36—N31	0.0 (3)
C4—C4A—C8A—C8	176.21 (15)	C351—C35—C36—N31	−178.46 (15)
C5—C4A—C8A—C8	−2.3 (2)	C33—C34—C341—O341	−114.67 (16)
C7—C8—C8A—N1	−178.14 (16)	C35—C34—C341—O341	64.32 (19)
C7—C8—C8A—C4A	1.7 (3)	C36—C35—C351—O351	102.07 (19)
C4—C3—C37—O31	−88.42 (18)	C34—C35—C351—O351	−76.3 (2)
C2—C3—C37—O31	92.08 (18)		

### (V) {5-[(2-Chloroquinolin-3-yl)methoxy]-4-(hydroxymethyl)-6-methyl-pyridin-3-yl}methanol Hydrogen-bond geometry (Å, º)

**Table:**

*D*—H···*A*	*D*—H	H···*A*	*D*···*A*	*D*—H···*A*
O341—H341···N31^i^	0.91	1.81	2.7098 (19)	174
O351—H351···O341	0.91	1.86	2.7299 (19)	158
C4—H4···O351^ii^	0.95	2.60	3.374 (2)	139

Symmetry codes: (i) *x*−1/2, −*y*+3/2, *z*+1/2; (ii) −*x*+1/2, *y*−1/2, −*z*+1/2.
